# Global Proteomics Revealed *Klebsiella pneumoniae* Induced Autophagy and Oxidative Stress in *Caenorhabditis elegans* by Inhibiting PI3K/AKT/mTOR Pathway during Infection

**DOI:** 10.3389/fcimb.2017.00393

**Published:** 2017-09-06

**Authors:** Arumugam Kamaladevi, Krishnaswamy Balamurugan

**Affiliations:** Department of Biotechnology, Alagappa University Karaikudi, India

**Keywords:** *Caenorhabditis elegans*, *Klebsiella pneumoniae*, proteomics, host-pathogen interaction, liquid isoelectric focusing, PI3K/AKT/mTOR pathway

## Abstract

The enterobacterium, *Klebsiella pneumoniae* invades the intestinal epithelium of humans by interfering with multiple host cell response. To uncover a system-level overview of host response during infection, we analyzed the global dynamics of protein profiling in *Caenorhabditis elegans* using quantitative proteomics approach. Comparison of protein samples of nematodes exposed to *K. pneumoniae* for 12, 24, and 36 h by 2DE revealed several changes in host proteome. A total of 266 host-encoded proteins were identified by 2DE MALDI-MS/MS and LC-MS/MS and the interacting partners of the identified proteins were predicted by STRING 10.0 analysis. In order to understand the interacting partners of regulatory proteins with similar or close pI ranges, a liquid IEF was performed and the isolated fractions containing proteins were identified by LC-MS/MS. Functional bioinformatics analysis on identified proteins deciphered that they were mostly related to the metabolism, dauer formation, apoptosis, endocytosis, signal transduction, translation, developmental, and reproduction process. Gene enrichment analysis suggested that the metabolic process as the most overrepresented pathway regulated against *K. pneumoniae* infection. The dauer-like formation in infected *C. elegans* along with intestinal atrophy and ROS during the physiological analysis indicated that the regulation of metabolic pathway is probably through the involvement of mTOR. Immunoblot analysis supported the above notion that the *K. pneumoniae* infection induced protein mis-folding in host by involving PI3Kinase/AKT-1/mTOR mediated pathway. Furthermore, the susceptibility of *pdi-2, akt-1*, and *mTOR C. elegans* mutants confirmed the role and involvement of PI3K/AKT/mTOR pathway in mediating protein mis-folding which appear to be translating the vulnerability of host defense toward *K. pneumoniae* infection.

## Introduction

Infection by *Klebsiella pneumoniae* is one of the most significant problems facing human health. Although several necessary measures have been taken to control the infections caused by *K. pneumoniae*, the emergence of new drug-resistance superbugs poses a great level of threat. In spite of the risk associated with *K. pneumoniae* to human health, it has been investigated to a minimal extent. Therefore, insightful knowledge on how pathogen alters the host cellular immune defense using a eukaryotic model organism may potentially open a new avenue for therapeutic interventions. An appropriate *in vivo* model system is supposed to not only satisfy the scientific community, but also the research ethics (Zak and O'Reilly, 1993). Moreover, it has also been considered that not all outcomes of pre-clinical studies using model animals could be effectively extrapolated to humans (Fuchs et al., 2009; Evans et al., 2010). Hitherto, there are several mammalian models with diversified physiological, anatomical, molecular, and genetic characteristics have comprehensively been utilized to understand host-pathogen interactions as well as in pre-clinical assessment of drugs (Means and Aballay, 2011). Of late, numerous reports have argued the extensive use of several alternative models such as insects, small vertebrates, and nematodes for studying bacterial infections (Kurz and Ewbank, 2007; Lopez Hernandez et al., 2015).

In the midst, *Caenorhabditis elegans*, a nematode model provides an excellent model to explore the cellular and molecular impacts in host by *K. pneumoniae*, particularly since we established it as a better surrogate host for *K. pneumoniae* infection (Kamaladevi and Balamurugan, 2015). Several studies on host-pathogen interaction using *C. elegans* against various bacterial pathogens provided insight knowledge on cellular and molecular immune signaling pathways that are conserved to mammals during infections (Kim, 2008; Marsh and May, 2012). Albeit, *C. elegans* has been extensively employed in forward and reverse genetic approaches to derive wide knowledge at the transcriptome level, the prominence of proteomic changes is still remains at its preliminary stage. Hence, exploring *C. elegans* with different pathogens complement abundant information to the existing transcriptomics data that will increases our understanding on host response to infections. For the past few decades, several key findings were discovered in *C. elegans* with high relevance to mammals (Grompone et al., 2012). Also, the shared common mechanisms between nematodes and mammals have been validated by using relevant *in vivo* model organism like *C. elegans* complemented with advanced tools in proteomics required addressing the host-pathogen interaction mediated complications.

The innate immune system is considered as a first line of defense against invading pathogens. Generally, it is an evolutionarily ancient and phylogenetically conserved from nematodes to mammals. Stimulation of innate immune system orchestrates several signaling pathways involved in defending the invading pathogens and protects the host system against infections (Akira, 2009). This response required an organized coordination between genomic and proteomic machineries. In *C. elegans*, host response against infection has been resulted in activation of complex network of pathways involved in metabolism, immunity, stress pathway, and aging (Singh and Aballay, 2006). This indicated that host defense is not only a single process but it is a process of complicated networks. Hence, analyzing the global level response either at the transcriptional or translational level will advance our understanding in host-pathogen interactions.

Hitherto several researches applied the combination of both transcriptional and translational tools to understand the host-pathogen interaction using *C. elegans*. Bogaerts et al., utilized *C. elegans* to decipher the defense mechanism of host at their protein level against *Staphylococcus aureus* (Bogaerts et al., 2010a) and *Aeromonas hydrophila* (Bogaerts et al., 2010b). The kinetic analysis of *C. elegans* proteome by using 2D-DIGE at different time-points revealed involvement of citric acid cycle and chaperon molecules in host defense against Gram positive bacteria *S. aureus*. Whereas, these regulatory proteins were regulated in an opposite manner in *C. elegans* infected with Gram negative bacteria *A. hydrophila*. Comparing the outcome of both studies they concluded that the host response is more specific to pathogens. Though data on proteomics advanced our understanding to next level, the connection between the regulation of proteins and genes against any infection process was not well-understood. Later in 2015, the connections between the transcriptional and translational regulations of C-type lectins and AMP-activated protein kinase were deciphered through genomics and proteomics analysis in *C. elegans* against *Bacillus thuringiensis* infection (Yang et al., 2015). Since characterizing the transcriptional and translational relationship is critical in understanding the host defense during host-pathogen interaction, we analyze the regulation of host regulatory proteins at their transcriptional and translational levels during *K. pneumoniae* infection. Previously, our group reported the role of few regulatory proteins and signaling pathway(s) involved in host defense of *C. elegans* against *Vibrio alginolyticus* (Durai et al., 2014), *Pseudomonas aeruginosa* (Balasubramanian et al., 2016), and *Protease mirabilis* (Jebamercy et al., 2016) using both proteomics and transcriptional tools.

With this background, the present study aimed to investigate the system-level overview of changes in host proteome using *C. elegans* against *K. pneumoniae* infection. Here we employed a conventional 2DE, liquid phase IEF, MALDI-MS/MS, nano LC-MS/MS, bioinformatics analysis along with molecular analysis (Western blot and real-time) to decipher the host defense in *C. elegans* against *K. pneumoniae* infection.

## Materials and methods

### Bacteria and culture condition

*K. pneumoniae* was obtained from American Type Culture Condition (ATCC). *Escherichia coli* OP50 was provided by Caenorhabditis Genetic Center (CGC), MN, USA. Both the cultures were inoculated in Luria Bertani medium and incubated at 37°C.

### Maintenance and synchronization of *C. elegans*

The *C. elegans* wild type and mutant strains were originally obtained from the CGC, MN, USA and grown on a solid nematode growth medium (NGM) plates containing *E. coli* OP50 as a standard food source. The *C. elegans* strains used in the present study were Bristol N2 (wild-type), VC2312 (*let-363)* mTOR mutant, BQ1 (*akt-1*), and VC858 (*pdi-2*). The age-synchronized L4 *C. elegans* were used in all bioassays. To obtain L4 staged *C. elegans*, the gravid hermaphrodites containing eggs inside the uterus were treated with commercial bleach solution containing 5 M potassium hydroxide in a ratio of 1:1 and the resulting solution containing eggs were transferred to the fresh NGM plates seeded with *E. coli* OP50. The plates containing eggs were incubated at 20°C for favoring the hatching. Thus, obtained stage-synchronized *C. elegans* were allowed to reach the L4 stage and used in all bioassays.

### Infection process and protein extraction

The age-synchronized L4 stage *C. elegans* were infected with *K. pneumoniae* for different time points (12, 24, and 36 h). Here, the nematodes fed on *E. coli* OP50 at respective time points were served as controls. After infection, the nematodes were washed thoroughly with sterile M9 medium to remove the bacterial contaminants. The samples were then flash frozen in lysis buffer [7 M urea, 2 M thiourea, 4% CHAPS, and 30 mM Tris-HCl, pH 8.5, and protease inhibitor cocktail (Sigma)] and stored at −86°C. When required, the samples were homogenized on ice for 1 min with 5 s pulses for every 5 s by employing sonicator. The insoluble cellular debris was separated by centrifugation at 12,000 rpm for 15 min at 4°C. The protein concentrations were determined with a Bradford assay Kit (Bio-Rad) using bovine serum albumin as a standard.

### 2D-gel electrophoresis

The 2D-gel electrophoresis was performed according to Balasubramanian et al. (2016) with minor modifications. A concentration of 1 mg of protein was taken in the volume range of (50–75 μl). The samples were processed to remove the non-specific biological contaminants such as lipids, nucleic acids, and salts using 2D cleanup kit (GE healthcare). The protein samples were dissolved in sample buffer containing urea and thiourea. Prior to 2DE, the sample containing total protein was loaded onto an immobilized pH gradient (IPG) strips (24 cm, pH 3–10 NL, GE Healthcare) and rehydrated for 12 h at room temperature. The rehydrated strips were subjected to isoelectric focusing (IEF) using Ettan™ IPGphor 3 isoelectric focusing system and accessories purchased from GE Healthcare. Then the strips were equilibrated in SDS equilibration buffer containing DTT (10 mg/ml) and iodoacetamide (25 mg/ml) for first and second equilibration respectively for 20 min each. The second dimensional separation of proteins was performed using 12% denaturant SDS polyacrylamide gels. After electrophoresis, the gels were stained with Coomassie Brilliant Blue (CBB) G-250.

### Image acquisition and analysis

The protein separated in SDS gels were digitized using Gel scanner-III and the scanned gel images were analyzed with the ImageMaster 2D platinum version 7 software (GE Healthcare). Briefly, after automatic spot detection, the manual editing of images like images like adding, splitting and removing of spots were done to increase the recognition of majority of spots. To match the spots across gels in each replicates, a class and match set were obtained with the available gel images. Among them, based on the gel and spot quality one gel was selected as the master gel for each time points, against which all other respective gels were matched. Spots, found in a match set member but absent in the master gel were added manually to the master gel. Then the automatic matching of spots on each gel was performed and matching across gels was manually verified. For each, match set, each gel was normalized to minimize the variability due to slight difference in protein load per gel, staining efficiency and image capture. This normalization was obtained by dividing the raw intensity of each spot in a gel by the total intensity of all spots in that gel that was included in the master gel. And all the analyses were performed on normalized quantities. The normalized values of all proteins were exported to in-built statistical tools for statistical analysis. The protein spots which had significant changes of >1.5-fold and *p* < 0.05 were considered as differentially regulated proteins.

### In gel trypsinization and identification of proteins

The differentially regulated proteins were identified by following the procedure of Durai et al. (2014) with minor modifications. In brief, the protein spots of interest were manually exercised from the SDS PAGE gel with end-remove pipet tips to accommodate the various diameters of spots. The gel pieces in the microtube was destained and dehydrated with 30% acetonitrile (ACN) and 100 mM ammonium bicarbonate in 1:1 ratio. After washing, the wash solution was removed and the gel pieces were lyophilized. The lyophilized gel pieces were further dehydrated thrice with 50 μl of ACN and subsequently reswollen with 50 μl of 50 mM ammonium bicarbonate for 10 min. Again the protein spots were rehydrated with Acetonitrile and lyophilized. The protein containing gel spots were subjected to reduction and alkylation by adding 10 mM DTT and 50 mM iodoacetamide, respectively. Subsequently, the traces of DTT and iodoacetamide were removed from the gel spots by dehydrating and lyophilization. Then the dried proteins spots were rehydrated in a protease solution containing 2.5 ng/μl of trypsin (Sigma) solution and incubated in ice for 45 min. The mixture was incubated at 37°C for trypsinization. After 16 h of enzymatic digestion and vortexing for 2 min at high speed, the peptides were extracted by adding extraction buffer containing 50 mM ammonium bicarbonate, 50% can, and 0.1% formic acid. The step was repeated twice to increase the yield. The extracts were pooled together and concentrated by using speed Vac. The peptides were dissolved in MS grade water and were desalted using ZipTipC 18 pipet tips (Millipore). About 0.5 μl of α-Cyano-4-hydroxycinnamic acid was mixed with equal volume of desalted samples and allowed to dry for 4–6 h. The MALDI TOF/TOF analysis was performed using MALDI-TOF/TOF analyzer (AXIMA Performance, SHIMADZU BIOTECH) in a positive reflectron ion mode. With peptide mass fingerprint (PMF) and MALDI TOF/TOF, the proteins were identified against the all entries of *C. elegans* database using the MASCOT (Matrix Science Version 2.2) as a search engine. During search, the carbamidomethylation and oxidation was set as fixed and variable modifications, respectively. In the MASCOT search, the mass tolerance of 1.2 Da with one missed cleavage per peptide was allowed in all searches. Proteins with constant hit against different mass tolerance and significant scores were considered. All the proteomics data have been deposited to the ProteomeXchange Consortium via the PRIDE (Vizcaino et al., 2016) partner database with the dataset identifier PXD007151.

### Bioinformatics analysis

The protein-protein interaction among the differentially regulated proteins identified at different time points were explored and the interacting-network map was created by using STRING 10.0 with a high confidence score of 0.7. The interacting partners were identified for their functions and annotated manually (Schmutz et al., 2013). The Gene ontology (GO) and the functional enrichment was performed using DAVID and KEGG pathway analysis. In order to eliminate the redundancy of proteins and to reduce the number of terms to a smaller meaningful set, the list of identified proteins were analyzed by ReviGO tool (Supek et al., 2013; Larance et al., 2015).

### Liquid-phase isoelectricfocusing (IEF) and LC MS/MS

The proteins isolated from *C. elegans* exposed to *K. pneumoniae* were separated by liquid-phase isoelectric focusing (IEF) with the MicroRotofor cell (Bio-Rad Laborotaries), according to their isoelectric points (pI). Approximately 2 mg of protein samples were mixed with IEF buffer [7 M urea, 2 M thiourea, 4% CHAPS and 0.24% Triton X100, 5% glycerol, and 1.6% ampholytes (pH 3–10)]. The focusing chamber of MicroRotofor cell was loaded with 2.5 ml of samples and the electrode cells were filled with appropriate electrolytes. The IEF was performed by applying a constant power of 1 W at room temperature. After 3 h of focusing, protein fractions were separated into different compartments (~200 μl) and were collected immediately to avoid the diffusion of separated fractions. For identification purpose, the collected fractions were trypsinized and analyzed using nano-RPLC coupled with an Orbitrap Elite Mass spectrometer (Thermo Scientific, USA). The digested peptides were dissolved in 2% ACN containing 0.1% formic acid and loaded to a C18 guard column (5 μm, 100 μm × 2 cm) (Thermo Scientific). The injected piptides were allowed to pass through a linear solvent system of 5–100% ACN for 80 min with a flow-rate of 300 nl/min and given voltage of 1.9 kV. The final MS data acquired were over the mass range of m/z 350–4,000 Da and visualized using Xcalibur software (version 2.2.SP1.48) (Thermo Scientific USA) (Ananthi et al., 2011). The data analysis was done using Proteome Discover software v. 1.4 based on sequest algorithm using a database downloaded from Uniprot. During search, the carbamidomethylation of cysteine was considered as fixed modification and methionine oxidation, N-terminal acetylation and phosphorylation (S, T, Y) as variable modifications. Here, the precursor mass tolerance of 10 ppm, fragment mass tolerance of 0.5 Da with 2 missed cleavages was allowed in protein identification.

### Western blotting and immunodetection

For blotting, nematodes exposed to *K. pneumoniae* for different time-points (12, 24, and 36 h) were washed thoroughly with M9 buffer and homogenized in ice cold Phosphate buffered saline containing a protease inhibitor cocktail. The soluble fraction of lysates was separated from the mixture by centrifugation at 12, 000 rpm for 5 min at 4°C. The final protein concentration of the samples was determined with Bradford solution (Bio-Rad). For each experimental run, equal concentration of proteins were taken and mixed with a SDS-sample loading buffer. The mixture was boiled at 100°C for 2–3 min. After heat-denaturation, the samples were subjected to SDS-polyacrylamide gel electrophoresis, followed by electrophoretic transfer onto the polyvinylidene fluoride (PVDF) membranes (Amersham Biosciences). Then the membranes were transferred to the primary antibodies solution (1:2,000) and incubated for 6 h at 4°C. After required time of incubation, the membranes were washed thrice with tris-buffered saline containing 0.1% tween-20 and transferred to the secondary antibodies solution (1:1,000) conjugated with alkaline phosphatase for 4 h at 4°C. Finally, the membranes were transferred to the developing solution containing nitro-blue tetrazolium (NBT) and 5-bromo-4-chloro-3-indolylphosphate (BCIP). The membranes were allowed to develop until the intense bands were observed (Kamaldevi and Balamurugan, 2016). The antibodies used in the current study are, AKT, pAKT, mTOR, pmTOR (Cell signaling), actin (sigma), PI3-kinase, pPI3-kinase, PDI-2, anti-rabbit and anti-mouse conjugated with alkaline phosphatase (Santa Cruz Biotechnology).

### Survival assay in mutant *C. elegans*

Approximately 10 worms were transferred to the sterile M9 buffer containing *K. pneumoniae* (20%, 3 × 10^5^ cells/ml), in a sterile 24-well culture plate and incubated at 20°C. The survival of the nematodes was monitored at an interval of an hour till it attained complete mortality. The nematodes that did not show any response to touch and lack of pharyngeal pumping were considered as dead. Here, the nematodes fed on *E. coli* OP50 were considered as control. Each experiment was performed atleast three times independently under the similar conditions.

### Microscopic analysis

The defect caused by the *K. pneumoniae* in the nematodes' intestine was monitored under microscope. The infected nematodes were washed thrice with M9 buffer containing 1 mM sodium azide (anesthetizing agent) and placed on a 2% agarose pad. The physiological changes with the exposed *C. elegans* were examined under inverted fluorescence microscope (Nikon, Japan).

### Quantitative and qualitative ROS determination

The reactive oxygen species (ROS) production in nematodes during *K. pneumoniae* infection was quantitatively analyzed by confocal laser scanning microscopy (CLSM). The nematodes exposed to pathogen were washed thrice to remove the surface bacteria and transferred to the liquid M9 medium containing 5 μg/ml of 2′, 7′-dichlorofluoresceine-diacetate (DCFH-DA). The content was incubated in dark for 10 min at room temperature. Then the nematodes were washed thoroughly with PBS to remove the excess DCFH-DA and placed the worms on an agar pad (2%) containing an anesthetizer (30 mM sodium azide). Finally, the slides were subjected to microscopic observations (Carl zeiss, Germany). The relative fluorescent intensity of 2′, 7′-dichlorofluorescein (DCF) in the nematodes represents the amount of ROS production in nematodes (Kamaladevi et al., 2013).

The quantitative measurement of reactive oxygen species was performed by (DCFH-DA) method (Scherz-Shouval et al., 2007). The nematodes exposed to *K. pneumoniae* for different time-points (12, 24, 36 h) were washed thoroughly with M9 buffer. The infected nematodes were homogenized and the concentration of proteins was determined by Bradford's method. An equal concentration of protein was mixed with 5 μM DCFH-DA in a final volume of 2 ml of Tris-HCl. The mixture was incubated at room temperature for 45 min in dark condition. The level of DCF produced by ROS mediated oxidation of DCFH-DA was measured spectroscopically at 530 nm, following excitation at 485 nm and the level of DCF was quantified against DCF standard. Results were expressed as nmol of DCF/mg of protein.

### RNA isolation, RT-PCR, and real time PCR

The total RNA was isolated from N2 wild-type and mutants exposed to *K. pneumoniae* for different time-points (12, 24, and 36 h) by TRIzol method. For RT-PCR, the oligo-dT method was followed to synthesis a cDNA strands. According to instruction of SuperScript III kit (Invitrogen Inc., USA), the CDNA template was generated from 100 ng of RNA isolated from nematodes. PCR was carried out to amplify from the cDNA by using forward and reverse primers. Quantitative real time (qRT-PCR) was performed by using the Applied Biosystem SYBR Green fluorescence dye. The expression of the candidate genes were normalized by *act-2*. The relative gene expression was determined by calculating 2^−ΔΔct^.

### Statistical analysis

The 2D-gel electrophoresis was performed at least three times from the biologically different samples for consistency of differentially regulated proteins and statistical significance of fold-changes. Real-time analysis and quantification of ROS were conducted in triplicates and the statistical analysis was performed by one-way ANOVA using SPAA software 10.0. Significant differences between the groups were calculated by Dunnet's multiple range test (*P* < 0.05).

## Results

### Kinetic analysis of changes in the host proteome during *K. pneumoniae* infection

**Figure 1:**
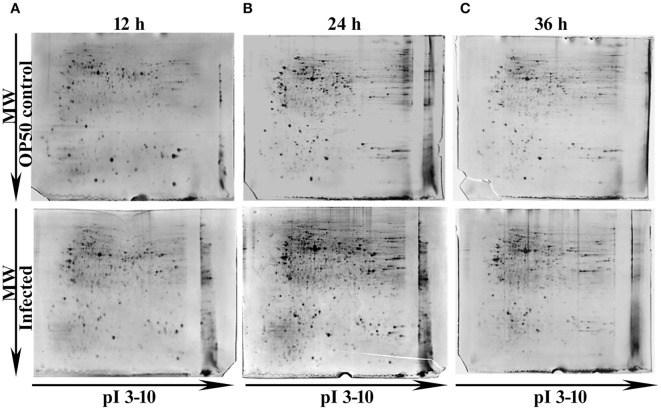
Representative gel images showing the detected protein spots at different time points **(A)** 12 h, **(B)** 24 h and **(C)** 36 h. The Upper panel representing the proteome of control nematodes fed on *E. coli* OP50 and the Lower panel representing the proteome of *K. pneumoniae* infected nematodes.

In our previous study Kamaladevi and Balamurugan (2015), we have reported that *K. pneumoniae* required 48 h for complete killing of nematodes. Hence, to understand the host response against *K. pneumoniae* pathogenesis at their proteome level, 12, 24, and 36 h were considered. Using conventional proteomic approach, infection induced host response against *K. pneumoniae* was monitored kinetically. The CBB stained gel images of respective time-points were compared and approximately > 300 protein spots were detected in each time-points. Figure 1 showed the CBB stained gel images of 12 (Figure 1A), 24 (Figure 1B), and 36 h (Figure 1C). The protein spots matched between the OP50 control and *K. pneumoniae* infected at 12, 24, and 36 h were 381, 630, and 742, respectively. We performed three biologically independent experiments and used the stringent criteria of a 1.2 Da mass tolerance and more than 1.5-fold regulation. This resulted in an identification of a total of 53, 82, and 43 regulatory proteins at 12 Supplementary Table 1, 24 Supplementary Table 2, and 36 h Supplementary Table 3, respectively.

### Protein-protein interaction and biological significance of the identified regulatory proteins at different time points

**Figure 2:**
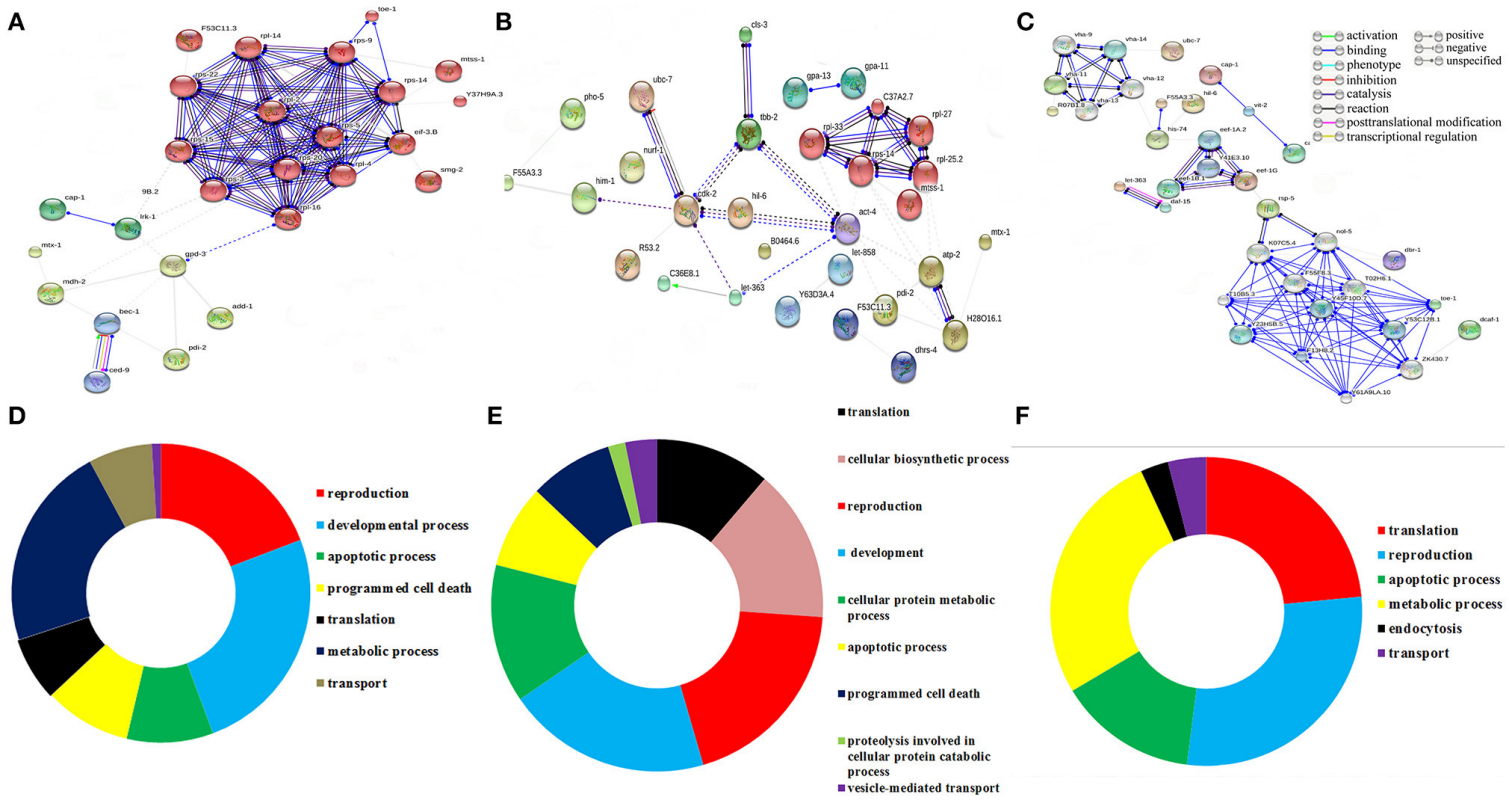
Protein-protein interaction of the proteins identified at **(A)** 12 h, **(B)** 24 h, and **(C)** 36 h using STRING 10.0 software and based on their function the identified proteins were classified using DAVID **(D–F)**. The interactive modes of STRING analysis results were available at their website 12 h (https://string-db.org/cgi/network.pl?taskId=2ewoymMJQSou), 24 h (https://string-db.org/cgi/network.pl?taskId=KS9tVO9Zw468), and 36 h (https://string-db.org/cgi/network.pl?taskId=iBw1EvsLlGVD).

**Figure 3:**
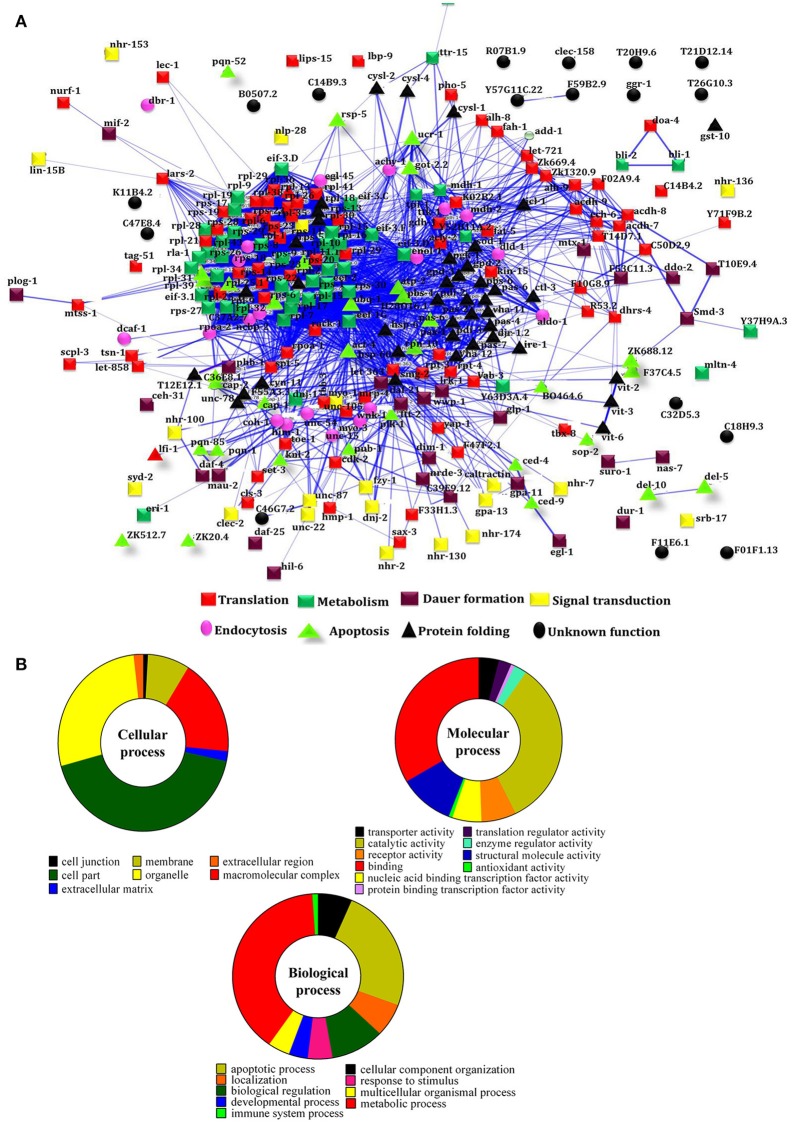
**(A)** Protein-protein interaction of all the identified proteins were manually annotated based on their function. The interactive mode of STRING analysis results was available at their website https://string-db.org/cgi/network.pl?taskId=I10REy8oLgGn **(B)** Gene ontology analysis for the identified proteins based on their molecular, biological and cellular functions

To decipher the interaction between the identified regulatory proteins and their role in biological activities during host defense, STRING 10.0 analysis was performed. The interactions between the identified proteins were analyzed based on the literature with a high confidence score of 0.7. The interaction between the proteins identified at 12 Figure 2A https://string-db.org/cgi/network.pl?taskId=2ewoymMJQSou), 24 Figure 2B https://string-db.org/cgi/network.pl?taskId=KS9tVO9Zw468), and 36 h (Figure 2C https://string-db.org/cgi/network.pl?taskId=iBw1EvsLlGVD) were analyzed separately and their biological functions were determined by Gene ontology (GO) database. The GO results revealed that the major group of identified proteins at different time points were predominantly encodes for metabolism, development, translation, reproduction, protein unfolding, and apoptosis (Figures 2D–F). Concomitantly, in order to investigate the interactions between the proteins identified at 12, 24, and 36 h and their biological significance in elucidating the host defense against *K. pneumoniae* infection, STRING analysis was performed with the total list of identified proteins and they were manually annotated (Figure 3A https://string-db.org/cgi/network.pl?taskId=I10REy8oLgGn). Based on their biological functions, the identified proteins and their interacting partners were classified into six major classes such as metabolism, translation, dauer formation, signal transduction, endocytosis, apoptosis, and protein folding (Figures 3A,B).

### Identifying the interacting partners with liquid phase IEF

**Figure 4:**
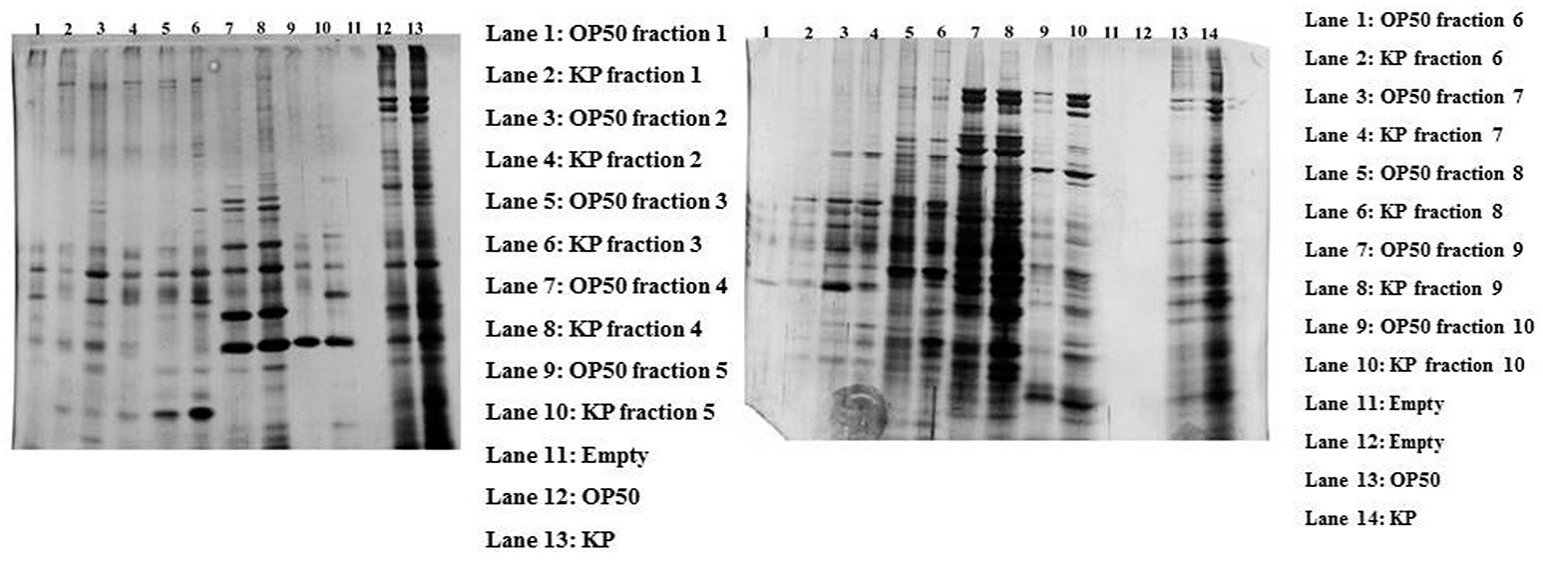
The gel representing the profile of proteins separated using liquid phase IEF. The control and experimental proteins were separated based on their pI using liquid IEF and the collected fractions were subjected to SDS-PAGE. The left gel shows the proteins present in the fractions 1–5 with an approximate pI of 3–6.8. The right gel shows the profile of proteins found in the fractions 6–10 with an approximate pI of 7–10.

In order to separate the interacting partners of regulatory proteins, a liquid IEF was performed. Proteins isolated from the control and experimental *C. elegans* homogenate were separated on the basis of their pI (pH 3–10) in solution. After the IEF separation, SDS-PAGE was performed to determine the separation of the proteins in all the 10 fractions Figure 4. Since most of the interacting proteins were identified to be in the pI ranges 5–9, the fraction numbers 5–8 were pooled together and enzymatically digested with trypsin. The peptides were analyzed by nano LC-MS/MS. A total of 88 proteins were identified in the IEF fractionations. The list of identified proteins was given in Supplementary Table 4.

### mTOR, a common player in all biological functions

**Figure 5:**
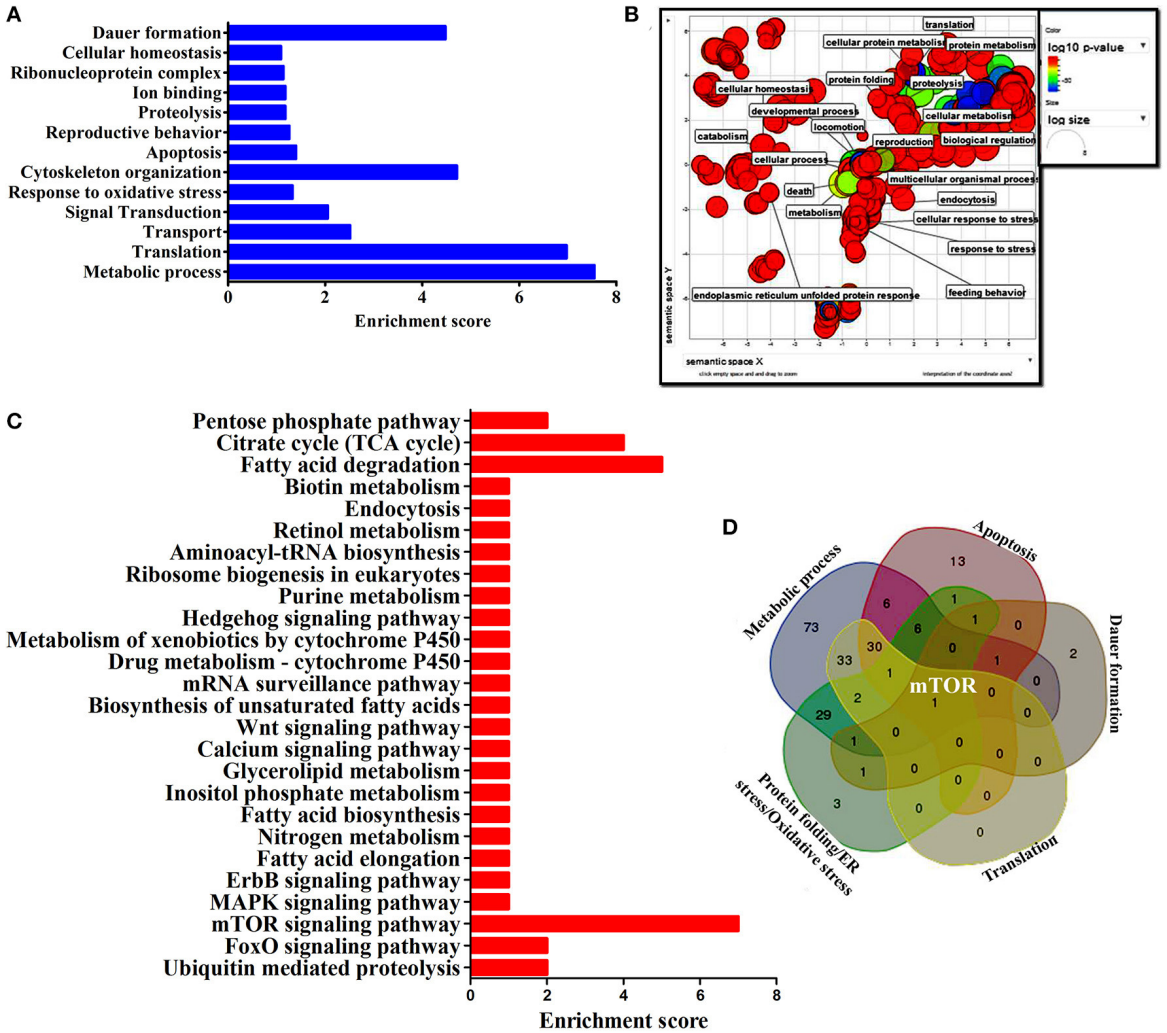
**(A)** The histogram representing the functional enrichment analysis for identified proteins based on their functions. **(B)** The enriched protein functional categories were defined by GO terms and the highly redundant functions were removed and the significant functions were plotted. **(C)** The histogram representing the enriched pathway based on the KEGG pathway analysis. **(D)** The Venn diagram represents the regulatory proteins which appears to be commonly involved in major biological functions.

To identify the protein that have major role in activating the host response against *K. pneumoniae* infection, several bioinformatics analysis were performed. The total of 266 proteins were enriched using the MetaCore software (DAVID) (Figure 5A) following enrichment analysis as described in the methods (Bessarabova et al., 2015). In proportion with the large number of regulated proteins in response to *K. pneumoniae* infection, more enriched GO categories were identified. In order to reduce the number of GO terms, enriched GO categories with false discovery rate (FDR) < 0.05 from DAVID analysis were submitted to the REVIGO tool (Supek et al., 2013). This tool uses the Uniprot as background database and has default semantic similarity measure (simrel). Investigation by this tool clearly showed that the biological process associated with metabolism, translation, protein metabolism, cellular homeostasis, protein folding, reproduction etc. were significantly over-represented among the proteins regulated in *C. elegans* during *K. pneumoniae* infection (Figure 5B). The lists of top 10 GO processes for these proteins in the host functions are provided in the Figures 2D–F. In addition, KEGG pathway analysis revealed that most of the identified proteins were regulated for metabolic pathway (Figure 5C). Concerning the GO process, the most regulated proteins were involved in metabolism, translation, apoptosis, development, immunity, and response to stimuli. Further, to identify the contribution of each protein in the different biological functions like metabolic process, translation, protein folding or oxidative stress, apoptosis and dauer formation, a Venn diagram were drawn with the list of proteins contributing to each function. The results revealed several overlapping proteins among the identified functions. Interestingly, a protein mTOR (mammalian target of Rapamycin) was found to have a contribution in all the biological functions (Figure 5D) and hence, was selected for further characterization studies.

### *K. pneumoniae* inhibits mTOR and causes metabolic stress

**Figure 6:**
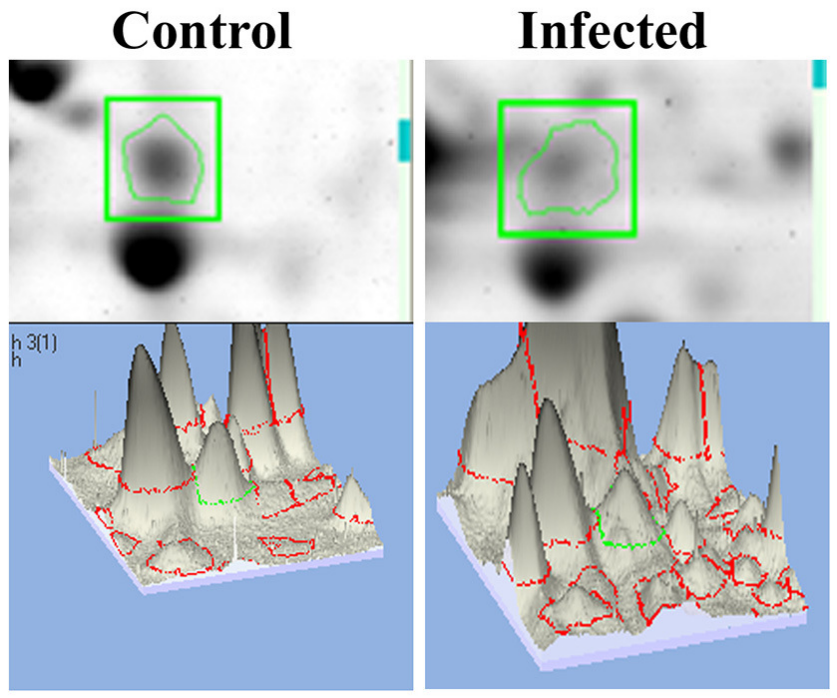
The representative spot showing the downregulation of mTOR and the 3D images of the control and infected samples confirmed their inhibition against *K. pneumoniae*.

mTOR, a mammalian Target of Rapamycin regulates several cellular process in normal system. However, the impact of bacterial infection on mTOR signaling remains unclear. In the present study, the proteomics approach coupled with bioinformatics analysis revealed mTOR has appeared to be involving in regulating the different biological functions. So we examined the regulation of mTOR in host during infection with *K. pneumoniae* in 2D gel analysis. Interestingly, we found that mTOR was significantly (*P* < 0.05) down regulated in 12 h when compared to the control. The downregulation of mTOR was showed in the 3D images Figure 6. The down regulation of mTOR was believed to induce a metabolic stress in host (Jia et al., 2004). mTOR is a major component for regulating the metabolic process (Tattoli et al., 2012). In *C. elegans*, metabolic stress acts as a double edged-sword (Kenific et al., 2010; Tattoli et al., 2012).

### Inhibition of mTOR induces intestinal atrophy and translation inhibition

**Figure 7:**
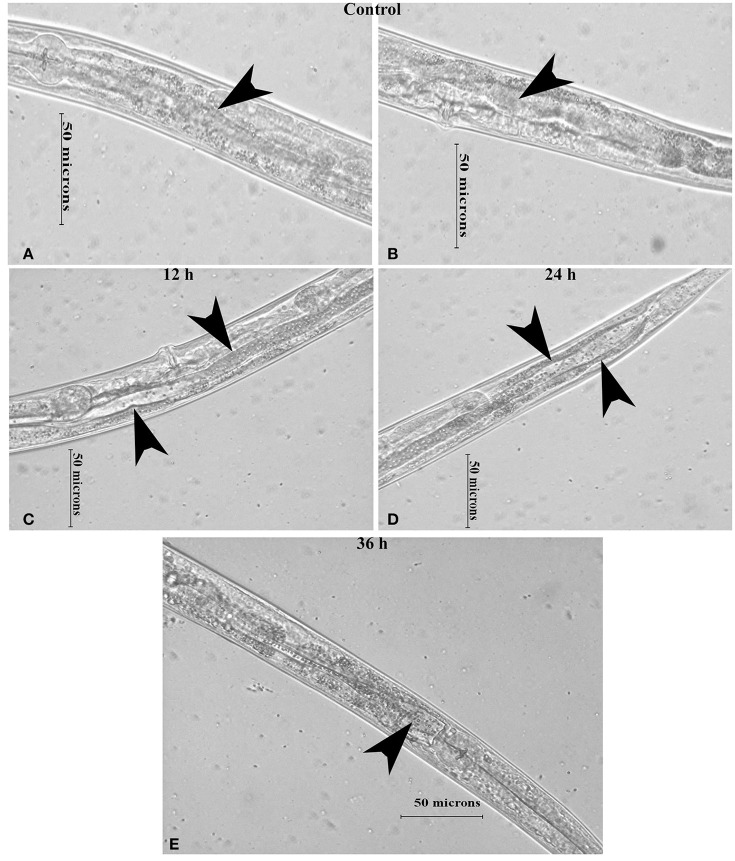
The micrographs representation the intestinal atrophy in nematodes exposed to *K. pneumoniae* at 12 **(C)**, 24 **(D)**, and 36 h **(E)** whereas the control **(A,B)** showed the normal intact intestine. The arrow head in the control panels indicates the normal intestine and the infected panels indicate the distended pharynx

**Figure 8:**
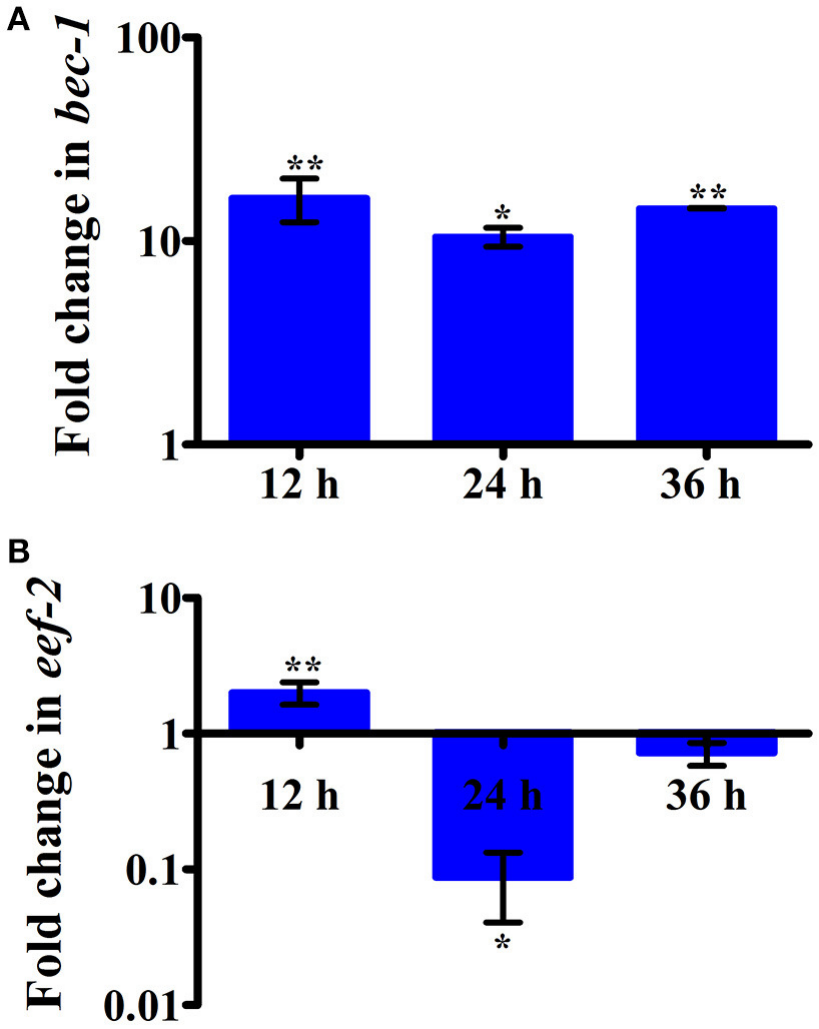
The histograms representing the upregulation of **(A)** *bec-1* which indicating the increased apoptosis in nematodes and downregulation of **(B)** *eef-2* indicating the inhibition of translation elongation factor in *C. elegans* against *K. pneumoniae* infection. Data are presented as mean ± *SD* of three biological replicates and the level of significance was analyzed by one-way ANOVA followed by Duncan's *post-hoc* analysis (^*^P < 0.05; ^**^P < 0.005).

Intestinal atrophy in a biological system was usually determined by the level of autophagy. It is well-established that autophagy is induced following the inhibition of mTOR in multicellular systems (Mortimore and Schworer, 1977; Chakrabarti et al., 2012; Kapuy et al., 2014). To examine the mTOR mediated autophagy, we monitored the physiological alteration in *C. elegans* intestine and marker for autophagy. The microscopic analysis of *C. elegans* exposed to *K. pneumoniae* displayed a distended intestine Figure 7. Due to the defective intestine, the infected nematodes could not absorb the nutrients from the ingested food. This promoted a dauer formation in infected nematodes. The results of short-time exposure assay indicated the presence of dauer nematodes during the course of infection (Table 1). This explicates that the infection with *K. pneumoniae* induced an abnormal dauer formation in nematodes. Interestingly, the dauer formation due to the deficient mTOR shares few unique and common features of normally formed dauer due to food starvation where it is not having severe intestinal atrophy (Long et al., 2002). Thus, the dauer formation along with intestinal atrophy in infected nematodes suggested that might be due to the downregulation of mTOR during infection. Furthermore, the increased autophagy was also confirmed at the molecular level by measuring the level of *bec-1*, a gene responsible for autophagy (Figure 8A). The upregulation of *bec-1* during infection suggested the activation of autophagic process during *K. pneumoniae* infection.

**Table 1:** Short-time exposure of nematodes exposed to *K. pneumoniae*.

**Time of exposure (h)**	**N2 wild type**	
	**Presence of OP50**	**Absence of OP50**
6	+	±
8	+	±
12	±	−

*+Nematodes were healthy with normal reproduction*.

*±Nematodes with reduced pharyngeal pumping, reproduction and dauer formation*.

*−Nematodes died*.

An inhibition of translation machinery is the other negative consequence of the inhibition of mTOR. The results of 2D gel electrophoresis also suggested that the infection with *K. pneumoniae* in *C. elegans* regulated several proteins that are encoded for translation. Hence, to investigate the status of translational regulatory gene in the *C. elegans* during infection, the mRNA level of *eef-2* was examined. The *eef-2* is the downstream regulator of mTOR, which is also an essential component for translation. The regulation of *eef-2* in the nematodes infected with *K. pneumoniae* was significantly downregulated during course of infection (Figure 8B). This result indicated that the downregulation of mTOR influences the regulation of *eef-2*.

### Role of mTOR signaling and PDI-2 in host response against *K. pneumoniae* infection

**Figure 9:**
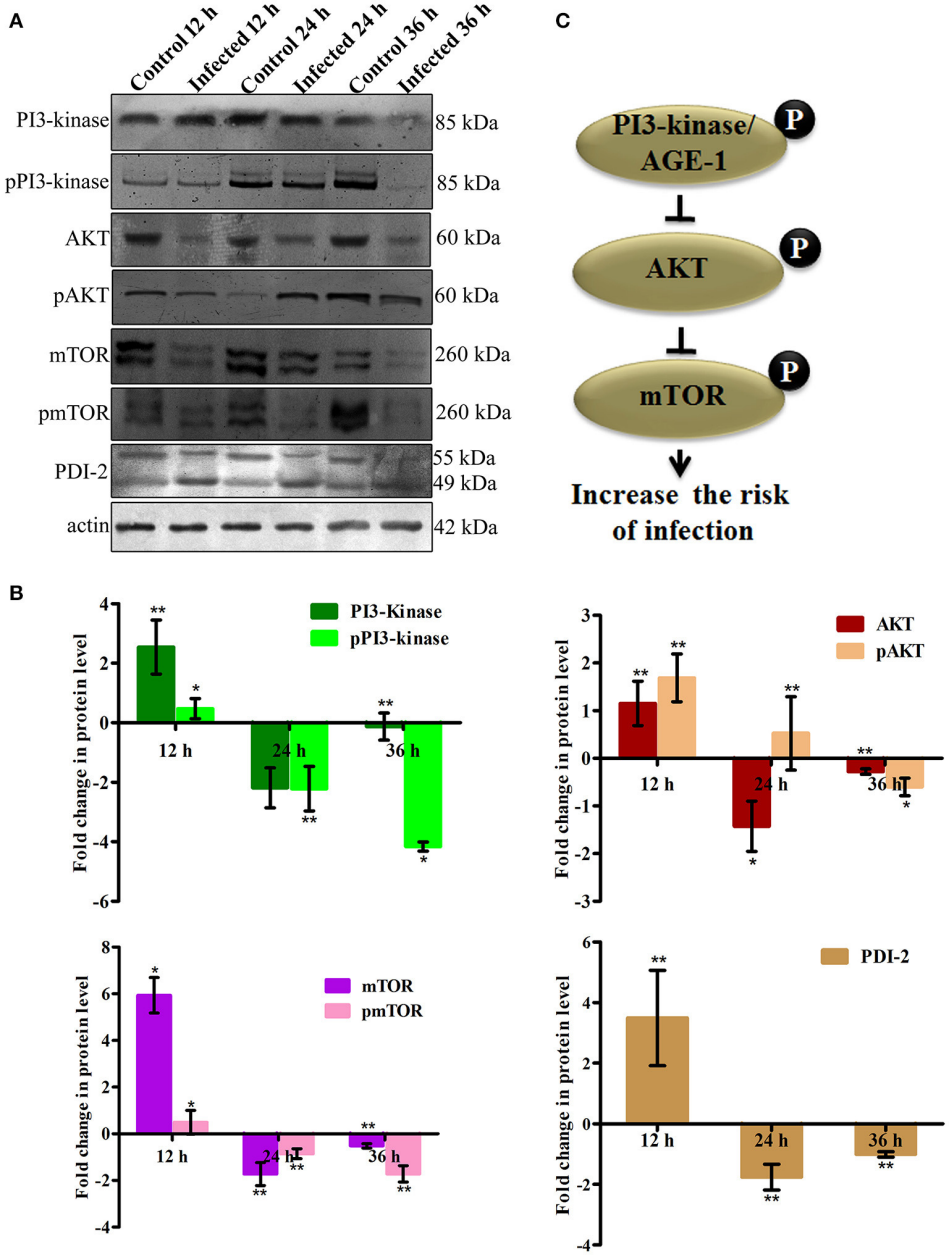
**(A)** The western blot analysis showing the regulation of upstream regulators of mTOR in *C. elegans* at different time points (12, 24, and 36 h). The downregulation of PI3-kinase, pPI3-kinase, AKT, pAKT, mTOR, pmTOR, and PDI-2 were observed during the *K. pneumoniae* infection in nematodes. The membrane containing proteins were transferred to the developing solution containing nitro-blue tetrazolium (NBT) and 5-bromo-4-chloro-3-indolylphosphate (BCIP) and allowed to develop until the intense bands were observed. **(B)** The histograms representing the fold change in the regulation of total and phospho PI3-kinase, AKT, mTOR and PDI-2 in *C. elegans* during *K. pneumoniae* infection. **(C)** The schematic diagram representing the PI3-kinase/AKT/mTOR pathway in increasing the risk of host susceptibility towards infection.

To elucidate the role of mTOR signaling in host defense against *K. pneumoniae* infection, the upstream regulators of mTOR were also examined for their regulation at their protein level using western blot analysis. The PI3-kinase and AKT were found to be the upstream regulators of mTOR. Hence, the level of expression of PI3-kinase, AKT, and mTOR were investigated. The PI3-Kinase activates the AKT and induced the phosphorylation of mTOR in normal conditions (Schmitz et al., 2008; Rafii et al., 2015). Our results showed that the level of PI3-kinase and AKT was inhibited in infected nematodes Figures 9A,B. This in turn inhibited the phosphorylation of mTOR Figures 9A,B. The inhibition of PI3-kinase/AKT/mTOR signaling pathway appeared to be increased the risk of infection (Figure 9C) (Rafii et al., 2015). Additionally, PDI-2, the protein encoded for protein folding was also examined for their regulation. The PDI is a protein-di-isomerase, which catalyzes the protein folding. The downregulation of PDI-2 (Figures 9A,B) indicated that the *K. pneumoniae* infection not favors the protein not to fold properly at 24 h in the host system. The inhibition of PDI-2 in the *K. pneumoniae* infected samples in western blot analysis suggested the possibilities of important regulatory proteins were not in fully or properly folded state in *C. elegans*. The result of the present study goes well with the previous study wherein the similar kind of regulatory event was observed in *C. elegan*s during infection with *V. alginolyticus* (Durai et al., 2014).

### Susceptibility of PI3K/AKT/mTOR pathway and PDI-2 mutants to *K. pneumoniae* infection

**Figure 10:**
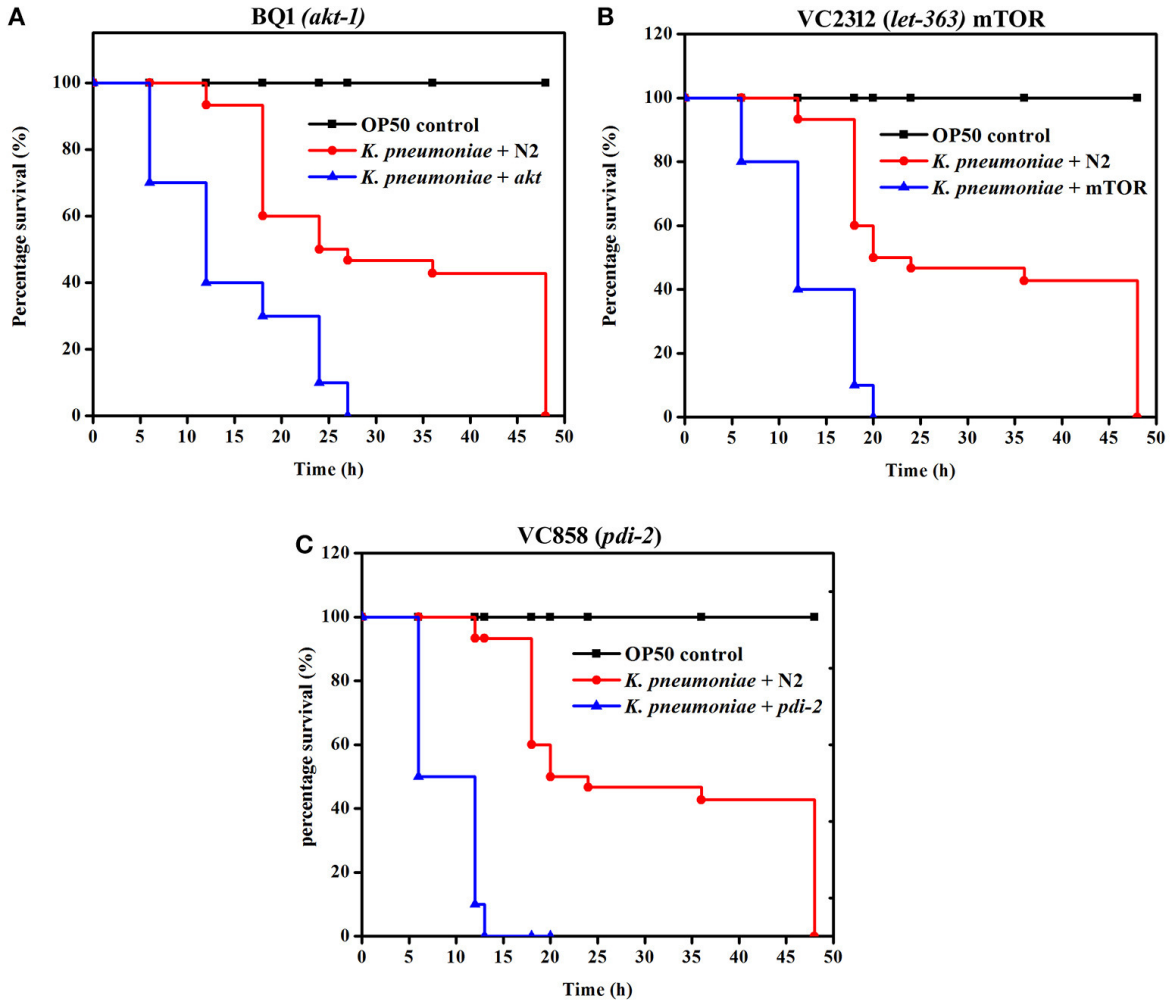
The role of PI3-kinase/ AKT/mTOR pathway and PDI-2 in protein misfolding was analyzed using the survival of **(A)** BQ1 (*akt*), **(B)** VC2312 (*let-363*) mTOR, and **(C)** VC858 (*pdi-2*) mutants.

To further confirm the role of PI3-kinase/AKT/mTOR signaling pathway and PDI-2 in host defense against *K. pneumoniae* infection, the *C. elegans* with specific mutation in AKT, mTOR and PDI-2 were examined for their survival during *K. pneumoniae* infection. The mutants of *akt* (Figure 10A), *mTOR* (Figure 10B), and *pdi-2* (Figure 10C) exposed with *K. pneumoniae* exhibited a significant (*P* < 0.05) shorter life span (mean lifespan of 27 ± 5, 20 ± 2, and 12 ± 7 h for AKT, mTOR, and PDI-2 mutants, respectively) than the controls (N2 mean lifespan of 48 ± 5 h) (Figure 10). The susceptibility of AKT, mTOR, and PDI-2 mutants to *K. pneumoniae* suggested the role and importance of PI3-kinase/AKT/mTOR pathway and unresponsive protein folding in *C. elegans* during host defense.

### *K. pneumoniae* infection induced oxidative stress in host

**Figure 11:**
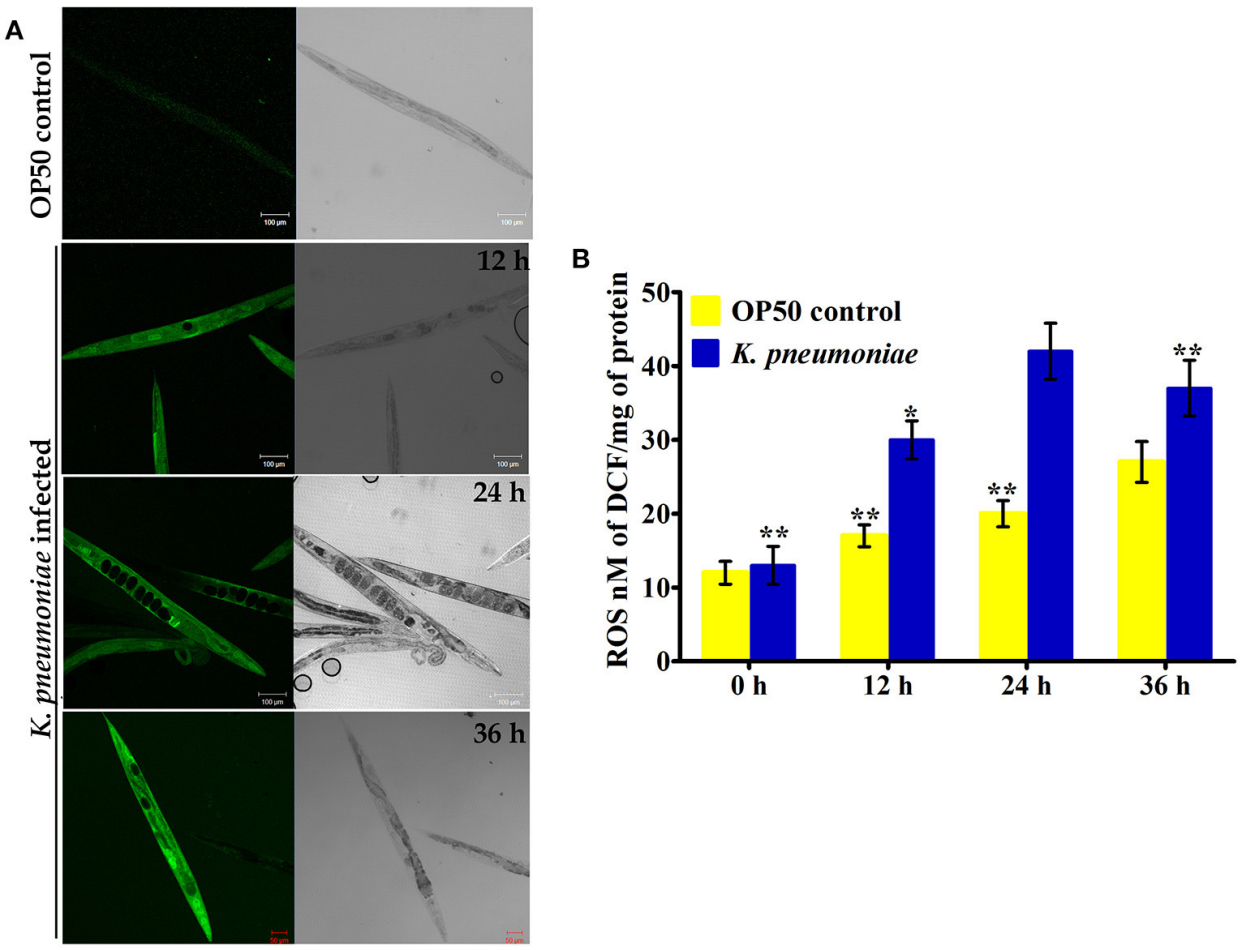
**(A)** The confocal laser scanning micrographs showing the ROS production in nematodes exposed to *K. pneumoniae* at different time-points (12, 24, and 36 h) by DCFH-DA staining method. **(B)** The histogram indicates the quantitative amount of intracellular ROS produced in host against *K. pneumoniae* infection. The values are expressed as mean ± *SD* of triplicates. Statistical analysis was performed by one-way ANOVA followed by Duncan's *post-hoc* analysis (^*^P < 0.05;^**^P < 0.005).

**Figure 12:**
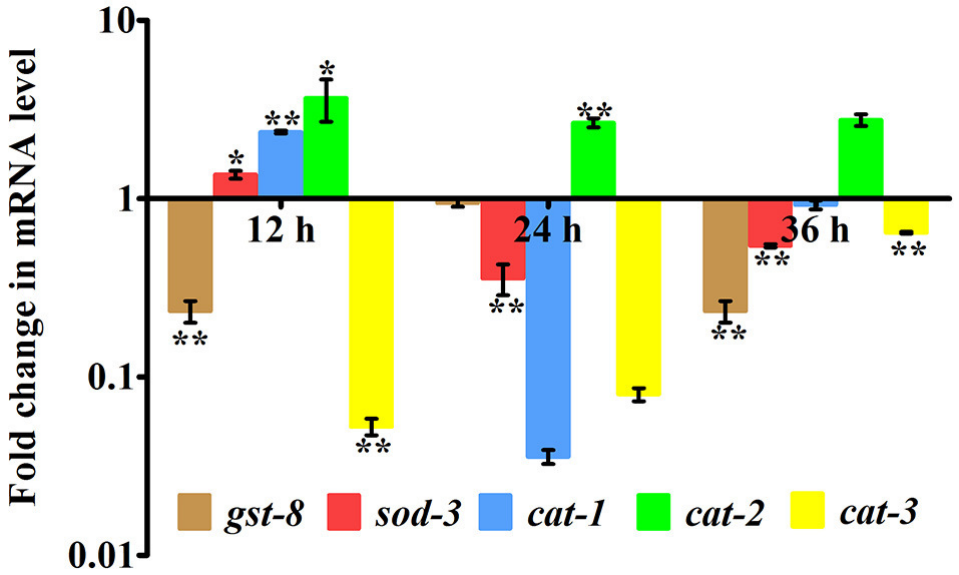
Quantitative real-time PCR showing the relative expression of the candidate antioxidant genes (*gst-8, sod-3, cat-1, cat-2, cat-3*, and *B-actin*) normalized over the relative expression of housekeeping gene, β*-actin* of respective time-points. Data are presented as mean ± *SD* of three biological replicates and the level of significance was analyzed by one-way ANOVA followed by Duncan's *post-hoc* analysis (^*^P < 0.05; ^**^P < 0.005).

In our proteomic analysis, we identified several stress responsive, detoxification, and protein mis-folding genes strongly induced after *K. pneumoniae* infection. The process of increased autophagy and translation blockage is also another source of oxidative burst (Chavez et al., 2007; Schmitz et al., 2008; Chakrabarti et al., 2012). The increased ROS is an alternate part of innate immune defense mechanism (Chavez et al., 2007). A significant increase in the level of intracellular ROS in the *C. elegans* exposed to *K. pneumoniae* than the control revealed the oxidative burst in host during infection (Figure 11A). Concomitantly, a high level of DCF fluorescence in the *C. elegans* with the increase in an infection time corroborated the generation of ROS due to infection mediated oxidative stress in the host (Figure 11B). Furthermore, in our 2D analysis, several proteins that encoded for antioxidants like *gst-8, sod-3*, catalase (*cat-1, cat-2*, and *cat-3*) were also found to be regulated. To validate these regulations, the mRNA levels of the identified proteins were determined by qPCR using specific primers. The mRNA levels of all the selected antioxidant enzymes were significantly (*P* < 0.05) upregulated during course of infection except *cat-2* (Figure 12). It appears that the levels of antioxidant enzymes increased to combat the elevated ROS during the *K. pneumoniae* infection in the host system.

## Discussion

*K. pneumoniae*, an opportunistic pathogen has been well-documented for its severity in causing community and nosocomial-acquired infections. Though it has been tagged as a most virulent pathogen, the lack of knowledge on host response against infection failed to eradicate their infections with available tools. Interestingly, *C. elegans* shares similarity to mammalian immune system, mainly signaling cascades in innate immunity toward bacterial infection (Alper et al., 2008). An inevitable characteristics of consuming microorganisms as a food has evident the selection pressure to evolve and maintain immune defense mechanism (Kim et al., 2002; Alper et al., 2010; Shivers et al., 2010). Although human lungs and nematodes' intestine vary in several aspects, it could be possible to characterize the virulence traits essential for infecting lung as well as respiratory tract, and also to understand the host innate immune response. Hence, the *C. elegans*, which is devoid of lungs system, has globally been accepted as a suitable model (Singh and Aballay, 2006; Akira, 2009) to study host response against lung and respiratory tract infecting microorganisms such as *P. aeruginosa* (Tan et al., 1999; Kirienko et al., 2013; Balasubramanian et al., 2016), *Streptococcus pneumoniae* (Bolm et al., 2004), *Streptococcus pyogens* (Jansen et al., 2002), *Microbacterium* Spp. (Hodgkin et al., 2000), *Mycoplasma iowae* (Pritchard et al., 2014), *S. aureus* (Bogaerts et al., 2010a; JebaMercy et al., 2011), *Legionella pneumoniae* (Brassinga et al., 2010; Komura et al., 2010; Hellinga et al., 2015), *Coxiella burnetti* (Battisti et al., 2017), and *Mycobacterium tuberculosis* (Galbadage et al., 2016). In addition, a study by Green et al. (2009) utilized *C. elegans* to characterize the impact of cigarette smoke on innate immune response of host fortifies the idea of using nematode as lung and respiratory disease model. In this scenario, the current study is aimed to demonstrate for the first time a detailed characterization of the host response or defense against *K. pneumoniae* infection using *C. elegans*. Here, the conventional proteomic method coupled with MALDI-MS/MS, LC-MS/MS, bioinformatics, and molecular approaches were utilized to decipher the involvement of regulatory signaling pathway(s) and other consequences in host during *K. pneumoniae* infection.

Earlier, the proteomic studies in *C. elegans* against different pathogens including *S. aureus* (Bogaerts et al., 2010a), *A. hydrophila* (Bogaerts et al., 2010b), *V. alginolyticus* (Durai et al., 2014), *P. aeruginosa* (Balasubramanian et al., 2016), and *P. mirabilis* (Jebamercy et al., 2016) exhibited the strong regulation of specific regulatory protein(s) at particular time-point. Such regulation depicted the specific role of particular protein in host defense. With this understanding, our previous knowledge on host-pathogen interaction between the *C. elegans* and *K. pneumoniae* at physiological levels, an early, mid, and late time-point of infections were considered for the present proteomics based investigations. In our previous study, we reported that *K. pneumoniae* required 48 ± 5 h for the complete killing of nematode (Kamaladevi and Balamurugan, 2015). Therefore, we opted to look at the changes in the proteomes at early (12 h), mid (24 h), and late (36 h) hours of infection.

A total of 266 differentially regulated proteins were identified at 12, 24, and 36 h respectively using conventional 2D method coupled with MALDI-MS/MS and liquid IEF coupled with LC-MS/MS analysis. The STRING analysis provided a network or interaction of the proteins identified at the different time-points. These identified proteins were subdivided into categories based on their biological functions which demonstrated that how the infection is anticipated at the proteome levels of whole organism at different time-points. The proteins which were identified, encodes for the biological functions like metabolism, translation, apoptosis, dauer formation, protein folding, signal transduction, and endocytosis. The functional enrichment results revealed that metabolic processes were enriched among the annotated functions. Furthermore, the mTOR was found to be involved in all identified functions. The mTOR is a key regulatory kinase that acts at the nexus of diverse nutrient-sensitive signals to regulate cellular metabolism and thus it controls the immune activation (Schmitz et al., 2008; Russell et al., 2011). The reduced feeding is the one of the prime factors that affects the nutrient signals. Previously we reported that infection with *K. pneumoniae* damaged the pharyngeal region of the *C. elegans* (Kamaladevi and Balamurugan, 2015). The affected pharynx attributed to the inhibited feeding and subsequently deprives nutrient signal during infection. The present study indicated that in 12 and 24 h of infection, the mTOR was downregulated during *K. pneumoniae* infection which clearly corroborated with earlier findings on deprived nutrient signals. The infection with *Shigella, Salmonella, P. entomophila*, and *P. aeruginosa* also reported to inhibit the level of expression of mTOR (Mohr and Sonenberg, 2012).

Nutrient starvation is known to inhibit mTOR, increase autophagy and inhibit translation (Jia et al., 2004; Watanabe et al., 2011; Chakrabarti et al., 2012). In *C. elegans* there has been an advantage of detecting the mTOR inhibition by physiological read-outs. The intestinal atrophy and dauer formation were the chief information to confirm the inhibition of mTOR. An intestinal atrophy is defined by the increased autophagy in the intestinal region of nematodes (Long et al., 2002). The distended intestine with an increased intestinal width corroborated the intestinal atrophy or increased autophagy in *C. elegans* by *K. pneumoniae*. Furthermore, the significant (*P* < 0.05) upregulation of *bec-1*, a marker for autophagy (Jia et al., 2009; Zou et al., 2014) in *C. elegans* confirmed the activation of autophagy in host during *K. pneumoniae* infection. From our 2D data, we showed that infection with *K. pneumoniae* regulated several proteins that codes for translation machinery. Among them, *eef-2* the gene which codes for elongation was studied previously to confirm the inhibition of translation machinery against *P. aeruginosa* infection (Balasubramanian et al., 2016). Similarly, the inhibited level of *eef-2* mRNA in *C. elegans* suggested that the *K. pneumoniae* infection affected the translational events in the host. Inhibition of protein translation reduced the synthesis of several immune genes which makes the host most vulnerable to an infection (Chakrabarti et al., 2012; Tattoli et al., 2012; Balasubramanian et al., 2016).

mTOR signaling, induces a Endoplasmic reticulum (ER) stress in host. The ER stress triggers the protein mis-folding and thus elevated the ROS level in host (Jia et al., 2004; Tattoli et al., 2012). An inhibited levels of mTOR and PDI-2 in western blot analysis suggested that the regulatory proteins are not folded properly in *C. elegans* during infection. Moreover, the elevated levels of intracellular and extracellular ROS in nematodes exposed to *K. pneumoniae* suggested the generation of oxidative stress in host against infection. Additionally, the significant upregulation of antioxidant genes corroborated the oxidative stress in host against *K. pneumoniae* infection. The elevation of oxidative stress is necessary to block the translation machinery during infection (Chakrabarti et al., 2012). Concurrently, the KEGG pathway analysis revealed that PI3K/AKT/mTOR pathway is responsible for host defense against *K. pneumoniae* infection. To confirm the role of this pathway against infection, the western blot analysis and survival assays using respective gene specific mutant *C. elegans* were performed. The inhibited activation of PI3-Kinase, AKT, and mTOR confirmed that *K. pneumoniae* arrested this pathway and the susceptibility of *C. elegans* having mutation in *pdi-2, akt-1*, and mTOR confirmed the above results. Recently, Rafii et al. (2015), through their clinical trials confirmed that inhibition of PI3K/AKT/mTOR increased the risk of infections in host. Therefore, present study deciphered that the inhibition of PI3K/AKT/mTOR pathway increased the susceptibility of host to *K. pneumoniae* infection.

**Figure 13:**
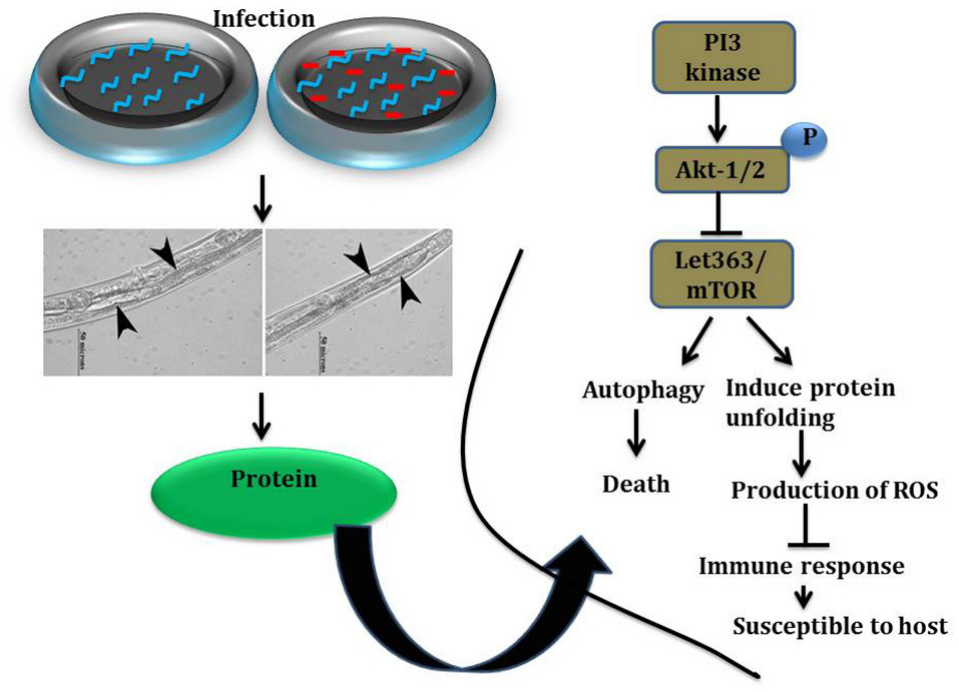
Graphical representation of host susceptibility to *K. pneumoniae* infection.

In summary, the data of the current study uncovered the critical role of PI3/AKT/mTOR pathway in the host response against *K. pneumoniae* infection in *C. elegans* Figure 13. The system-level over-view of host proteome during *K. pneumoniae* differentially regulated the proteins that encodes for metabolism, translation, signal transduction, apoptosis, protein misfolding, and dauer formation. Inhibition of mTOR, followed by subsequent changes in host cellular activities and hyper-susceptibility of PI3 kinase, *akt-1, mTOR, pdi-2*, and *eef-2* mutants unveiled the role of PI3K/AKT/mTOR pathway, protein folding, and translation machinery in host defense against infection. Moreover, the importance of host metabolic perturbations in infected host in addition to severe defects like autophagy, dauer formation, protein mis-folding, and oxidative stress advance our understanding in the multiple aspects of the host response against *K. pneumoniae*. These results will pave a way to the advancement of new therapeutic strategies against *K. pneumoniae* infection.

## Author contributions

AK and KB conceived and designed the experiments. AK performed the experiments, analyzed the data, and prepared the manuscript.

### Conflict of interest statement

The authors declare that the research was conducted in the absence of any commercial or financial relationships that could be construed as a potential conflict of interest.

## Supplementary material

Supplementary Table 1 List of proteins identified in nematodes exposed to *K. pneumoniae* for 12 h by MALDI-TOF/TOF analysis.

Supplementary Table 2 List of proteins identified in nematodes exposed to *K. pneumoniae* for 24 h by MALDI-TOF/TOF analysis.

Supplementary Table 3 List of proteins identified in nematodes exposed to *K. pneumoniae* for 36 h by MALDI-TOF/TOF analysis.

Supplementary Table 4 List of proteins separated by liquid phase IEF and identified by nano LC-MS/MS analysis.
