# Supplementary material for: Global Proteomics Revealed Klebsiella pneumoniae Induced Autophagy and Oxidative Stress in Caenorhabditis elegans by Inhibiting PI3K/AKT/mTOR Pathway during Infection
Source: Front Cell Infect Microbiol. 2017 Sep 6;7:393. doi: 10.3389/fcimb.2017.00393 (PMC5592217; doi:10.3389/fcimb.2017.00393)
Supplement: Supplementary Table 1 — List of proteins identified in nematodes exposed to K. pneumoniae for 12 h by MALDI-TOF/TOF analysis. [file Table1.PDF]

Spot No	Wormbase ID	Protein	Gene	MASCOT score	Peptides identified	No of values matched	% sequence coverage
57	WBGene0000692	Vitellogenin-2	*vit-2*	55	TFEPKTDYHYK NEIEKSI NFDKKVNGEKLVRIFRF AESRMLA LWRVAIVCSK ESTTVVRI QTGIKIQAVA GMKEQGYKSVI SLKVKV DGKSDL VEKFTCLQRHN R	24	7
267	WBGene0000197 8	Humpback-1	*hmp-1*	72	RAIAT NSGRFLILADSI DVK QTAPLLYTST RHPEHE EAR VTFSEYGR TK TAQENMET YK QAVR ILKASALANR RPNANSVRASERRPL QPAK RHQ QR	16	10
253	WBGene0000285	Electron transfer flavoprotein-ubiquinone oxidoreductase	*let-721*	77	MRISGVTLFRVCVVEKA LDELIPNWKGMEFHAKE FQKYKTHPSISKDTYVV KELKATRLIPVKDAK	12	11
242	WBGene0000372	Nuclear hormone receptor family membrane -130	*nhr-130*	71	FQFDRRTFGMSRLALGLN LVRDQPAGSHDVQLVTK LGKLALTATSRTIELDYS WFTKYPK	11	13
312	WBGene0000295	Pre-mRNA-spilicing factor	*let-858*	69	SKETRSPVRSRTRTGGAY IPPAKNLVQIVRSKGLL CR LRAILMETERSENALDR LDPEFEKLLKMKILADM K MTEEDTTSSGRGMLDQ LK EDSGERRSEERGGREVES	19	12

					DDR		
27	WBGene0000798	Uncharacterized protein C36E8.1	*C36E8.1*	65	RSTANAPKTETLVKRIPG SVIERALLKCVKNLILMQ KYIPASISKVLPKEKDML ETIRCFSAITR	15	9
32	WBGene0000369	Protein disulfide-isomerase 2	*pdi-2*	126	AATQLKEEGSDIKLGKT G PVAKPLADADAVKSEIE LKGEGIVLFKKLTQDGLK TWIQANRSHNLLFVSKIM EFFGLKLISLEEDM TKNP VKILVGKQLAPTWDKIQ SFPTIKEGAGASEEEK	15	24
325	WBGene0000091	Dauer Abnormal formation protein 25	*daf-25*	104	TTTEEAPKT KEQAAQR LLMDSGARMYLVNGIGK YPDSLKILYVIDRRALDV MLRFDWPIHKKVCSFLK	12	17
190	WBGene0000654	T-box transcription factor tbx-8	*tbx-8*	43	KNEMIVTKMFPKL EYVV RYKFSSGEWVKSGKAEK FNPFA K	8	12
333	WBGene0000168	Glyceraldehyde -3-phosphate dehydrogenase 3	*gpd-3*	55	TKPSVGINGFGRIGRLV LRLWRDGRLEKPAS LDD IKA AADGPMK	6	12
279	WBGene0000442	60S ribosomal protein L13a	*rpl-16*	64	KLLQ GDKYMSFLRGNE L KSLHAPSASRFRFCVVGR NAAPKIAQYQK	9	23
228	WBGene0000025	Cuticle collagen bli-1	*bli-2*	92	EAEDNDRTIQG FRAPYKP TRQPSGGYDSDGQTP PSS PRGRPGPPGFPGQDGPRG LRAEEPRPRRPPPPHRQT PHELY PEEQPYVREYVPE	12	14

					APRPRQGYEHSSGYGGD DRNQPEYEDISKPEEDK SQELRNHYYEKFLV		
292	WBGene000067	Transcription factor unc-3	*unc-3*	73	AHFEKHPPNNLRNGIHY RLSLMFQ NGIRQAITYEG QDKNPEMCRVLLTHEV MCSRCCEKKSCGNRYPG D PER	12	14
261	WBGene0000088	Peptidyl-prolyl cis-trans isomerase 11	*cyn-11*	44	VDRHGREILFNGRAMTD F KCIGSG ALVEAVRGAL DTDNKSLN LADNSIRKS AVRSQRTYHHLRFGRPFR SG ALLMLKNGSRSRTSSSAS HRTTGDISKAAADKWK QIVLLKQLVGSKFVAEEK	4	18
256	WBGene0001257	coiled-coil and C2 domain-containing protein 1	*Y37H9 A.3*	39	IL HHRRQQR FQKMAEK TEGNERKMKMNMRYLV EMKGFDKFHYEMR	14	5
346	WBGene0001031	Uncharacterized protein F59B2.9	*F59B2. 9*	78	RTMNRIFDKVEELFLRSA RWMT RNYIFTMKLENLP KTLGPLYKLLSNPNLVYT EFLETYKVLKSPSTGQA FR	10	16
360	WBGene0000352 6	Zinc metalloproteinase nas-7	*nas-7*	49	LLPWIITIVTVIPATLGHR NRVDLSKTSYYGQPYDY KSILHYDSLAFSKNGFPT MLPKVK	5	15
173	WBGene0000072	Adducin-related protein 1	*add-1*	60	E RPYYRAHPMVR	12	7
78	WBGene0000029 2	F-actin-capping protein subunit alpha	*cap-1*	78	AIQRAFRNTNSLKRNGT VDWRLALLRKAIKENNR KYVDMVK	8	16

328	WBGene00016978	Uncharacterized protein C56G2.3	*C56G2.3*	50	MSDEAWKE ALSAARYV KWKMQMLRREEERR	6	9
44	WBGene00003606	Nuclear hormone receptor family membrane	*nhr-7*	71	QSYTNRNKYVCRCLLVG MNPDYV RPDREKFSVE DKVIKHSRNLASRIRIPLE MTK	11	10
175	WBGene00003136	Maternal uncoordinated protein	*mau-2*	51	ALLGMAEALRHEITT TR GFPALSNKMELYFRIAKA H LECIKDFYVCTKYYHLVI KDMLEWSNRGVRPTVD W SKI LLAAGYDPK	10	12
286	WBGene00000066	Actin-4	*act-4*	80	CDDEVAALVVDNGSGM CKA GFAGDDAPRA VFPS IVGRPRVAPEEHPVLLTE AP LNPKS YELPDGQVITV GNERFRIIAPPER	10	21
365	WBGene00006819	Unc-87	*unc-87*	68	QSEALERWTLAQL RQTD GIIPSQ AGWNKNTNTRV KYCSQRGMTGFGSGRGM T GFGTARMTSFGAPRIWE LEYPEEAEISLDR	13	15
150	WBGene00009559	Metaxin-1	*mtx-1*	53	FVD ILK CGQDVV IDADL TTIEKNDTEILKQQANRK MWQSR DKE AAK	9	15
81	WBGene00004880	Regulator of nonsense transcript 1	*smg-1*	63	MDDSDDEYSRKDQVVVI I CRSVMESRFDLPRRLNVA ITRAKYGLVLVGNAKAT IRTKFGIKRRGDQRHHH HR	12	6
344	WBGene0000472	Sax-3	*sax-3*	68	MFNRKEQVNSHRVNSGK	16	7

	9				NGKKNEPMPVTRAALKV<br>TTKM DMAIAEKRLTSEQ<br>LIKWRGPPRIRMLNLTTL<br>RSSRASDGRRTPPNKNLG<br>GRPLK		
307	WBGene00013122	IMPACT	*Impt-1*	52	QLVFKFDRILKSNTKICR<br>MLELM DRMKHIN NLTR	5	13
184	WBGene00000900	Cell surface receptor daf-4	*daf-4*	53	RMDN EEDEVISIEC VYY<br>DEMECEKAVGCLAVFGL<br>PTQEINSTEPYLKSLGCM<br>PY HADS MNCENESSCR<br>DSDNSALLWASTPSNM D<br>LESLDKWYKSIVE FVCA<br>EKLHQTDEPPNYQMPFQ<br>VIGFD PTIGLMRVWNH I<br>MSSPDSSEG YHSGSSMK<br>N	6	16
327	WBGene00006832	Degenerin-like protein unc-105	*unc-105*	62	L RRPASIESTM SSRTKPR<br>F ENAPFPSITICNLNPYKK<br>TEGIAAA LSATGGLHAK<br>VRITVHDKNTTLWLGEL<br>YSK GKLSVMDIRIMKAL<br>CPVHGYMVR	11	10
271	WBGene00020897	Degenerin-like protein T28D9.7	*T28D9.7*	52	MAERLNSRASLGSSMGR<br>Q<br>FLDAFKSW DMDAVKIRE<br>EGTASTCLP PCKAYEMQ<br>ARYMS VGDLKSRTMTG<br>DFSRMDRMLSSFKSQSY<br>QR	13	8
216	WBGene00006704	Probable ubiquitin-conjugating enzyme E2	*ubc-7*	62	EQSSLLLKK QLADMRDY<br>PQKPPKM K	6	19

230	WBGene00022581	ZC262.4	*ZC262.4*	72	MNADKFVETT ISDFMRL NSSRDFEYFVSIA K YGEMLGTVGEKINLTAS APM ATDPSESSC	7	40
298	WBGene00001857	Histone H1.6	*hil-6*	59	APK ANATKLGDQVK ALVQTVGTGATGRAATG EKVKIAKPAAKKKTAA LK	9	25
229	WBGene00003162	Probable malate dehydrogenase	*mdh-2*	68	TLVQAAANSGLRFANAL VRGIKGEKVSAYEQKLI DASVPELNKNIAKGVAF VK	7	15
287	WBGene00021629	CTD small phosphatease-like protein 3	*scpl-3*	82	MSNVTPKCPALPVKLRP HLRTFLSRNHIRHRDLTIL GRNDTELLKYRLR	9	17
41	WNGene00015262	B0563.5	*B0563.5*	62	MTSTSVYFNDYYNRASS SKLELCMSPSSSTISKSHS LDE IPDVSARRKPEE STIGMDAPKK	6	53
227	WBGene00013847	CLASP-3	*cls-3*	72	DTSPT RRIRSPLKTP TIE AHDKLSEWLRLEEQSTS R LMEKIQNVQDLLQKSRSE NNKKMCLR	16	8
12	WBGene00001608	R07B1.8	*R07B1.8*	66	MTPHFTKL PKAMTNLIQ V YYVNR	10	12
275	WBGene00001225	Eukaryotic translation u=inititation factor 3	*eif-3.B*	75	QLEAPSDH WTTPEGND PELAGDADKADSLRI WDVRTQLPTWPFFRWSF DEKYFAC LKLEREQKLAF YTMRFKLDAGVHFNEVQ F APKASQEVVEKRKI MA AFDIIRSREQLDATRDER	14	13

318	WBGene00003526	Zinc metalloproteinase	*nas-7*	82	LLPWIITIVTVIPATLGHR NREELFGKHIPVEVVNDF KNGVSRAAKTCIRFVPR SILHYDSLAFSKNGF PTM LPKVKSATIGNAR	10	22
138	WBGene00022739	Heat repeat-containn=ing protein 1	*toe-1*	72	HLTVEKRLS NETAHRIG VAGLEQMKWDKVE WAL NEMAQRKVEDDVEQFVL EIVKVVGVGGVKILEH PEKIAQAIA RTAQT LPRN A LPLGKRMSYKMCEKLD ENLTGNVLLLAGELIRRG HRLSLIRRVLPAHIVK	18	9
335	WBGene00009973	F53C11.3	*F53C11.3*	72	AIATTFAHLGASVAIAAR RSSTGGVCEPFQMDVK CIQQKRAGVENMTK SLA SEWAKDSGDAMKASVP VGR	9	22
43	WBGene00022126	Phosphatodatecytidyltransferase	*Y71F9B.2*	76	MDEYRSEKMVDFVIVTK LMG PKMIEKIKYGVISYE NVKQDMCDLVTENRR LSVMSPAKMSKFLK	9	19
42	WBGene00009364	F33H1.3	*F33H1.3*	58	SGERYRYCNLDETTSK VLTDSINKREDAELKK NNQLLAPA MIKRETVK QAAP VQRRPEVQK	11	21
144	WBGene00023497	Lin-15B	*lin15B*	54	MQTLKNAIYLRKSVNSY SFSNVKRMQLQKEFAY YRGASVDVISQ SMIETLN NTA SRTT ASSQGPSSYP RKL PTSQSSSPST ATSAQ ARQNFVHKVTMEFQKRT YICRILHDFAERNNHLRS	17	9

					EER LAR		
301	WBGene0004415	60S ribosomal protein L4	*rpl-4*	58	YEATQSQ IRLPAVFRQAH AVNTKSGQGAFGNMCRN VNIAQKLA PGGHLGRGW SVP LPIMANSDFSRLNPY ASILR	11	21
277	WBGene00000423	CED-9	*ced-9*	77	TM ATGEMKEFLG IKVM GTIFEKKTVGNAQ TDQC PMSYGRNLFVYTSLFIKT RSWDDFMTLGKQ MKED YERMMFSLK	14	27
170	WBGene0019800	Single-stranded DNA-binding protein	*mtss-1*	76	SLSTISKSTVRCMSLTSK MAAEQPSKHAVSVFGKL MVQGRTQRNTYIIAQTV QPLA R	10	33
