# Supplementary material for: Global Proteomics Revealed Klebsiella pneumoniae Induced Autophagy and Oxidative Stress in Caenorhabditis elegans by Inhibiting PI3K/AKT/mTOR Pathway during Infection
Source: Front Cell Infect Microbiol. 2017 Sep 6;7:393. doi: 10.3389/fcimb.2017.00393 (PMC5592217; doi:10.3389/fcimb.2017.00393)
Supplement: Supplementary Table 2 — List of proteins identified in nematodes exposed to K. pneumoniae for 24 h by MALDI-TOF/TOF analysis. [file Table2.PDF]

Spot No	Wormbase ID	Protein	Gene	Mascot score	Peptides identified	No of peptides matched	% of sequence coverage
88	WBGene0000754	Nuclear hormone receptor family member nhr-153	*nhr-153*	78	MTPPQKFENGEK NQIL GKLIKIETKLEKVH DNGMPMGFLD QRNGP LIEKSFLQNTLISFMTN NVDKLI HSQK	9	17
576	WBGene0001980	Single-stranded DNA-binding protein, mitochondrial	*mtss-1*	71	CMSLTS KMAAEQPSK HAVSVFGKLMVQGRD E QGNRENPDQH	8	24
405	WBGene0002006	C-type lectin domain-containing protein 158	*clec-158*	68	FILSAFVVALVAADCA KNSMT GLWCVKGGM IVDGHRLK QI ESCLQL AISQ FKSR	5	35
285	WBGene0000670	Probable ubiquitin-conjugating enzyme E2 7	*ubc-7*	83	EQSSLLLK QLADMRR AILDFP RDYPQKPPKM KENYAEFK VAQCVRR	8	28
279	WBGene0000258	Target of rapamycin homolog	*let-363*	70	ITE NR YANYLLKML NNGNGLDEDT VKLAASH LSR D DLDR QQTIVE SVTARLVLE R MIMMRIPK KVT SDKR MIA QLAKQSPK LIEVC R EDI SVK NTAL STVLHMTQQV DVSAYAPR RGVDQ K AQMSR YG VEEK EMSV LKK ALG AYELEEKK AMGLIEK LIM LR FSS MCRKQGKNSM SR TKESR NAGTVLEK YSIVALSK EYR EK EKFPE R LVE GMKK K DTGGR	48	8
473	WBGene0000372	Nuclear hormone receptor family member	*nhr-136*	68	MCR ACREIMIK VQGI IGKLTVMELKYG	10	12

		nhr-136			EA GTKYQASMQR DFH LL AKDAV RYTR		
423	WBGene0000955	Accepts electrons from ETF and reduces ubiquinone	*mtx-1*	67	FVD ILKKALRL LAGKNDTEIL KKMWQ SRISK	8	9
267	WBGene0000444	60S ribosomal protein L35a	*rpl-33*	66	MADTAVARRP SAPTT GRLEGVFNKTGHTVAT R TRAIWGK	6	31
354	WBGene0001756	D-aspartate oxidase 2	*ddo-2*	72	AIPN AKITVLHDKP FKYVPYLKNLLLEQK D FTTFTIPKEH SVVVG STKQD NRWDLEITDE DREWSALRP GRK	11	21
464	WBGene0000653	Tubulin beta-2 chain	*tbb-2*	82	IDVYYNEANNGKYV P RAVLVDLEP GTMDSV RKLAVNMVPF PRLHFF MPGF APLSAKY LTVAA MFRNSSY FVEWIPNNV KISEQFTAMFR	15	19
293	WBGene0000362	Nuclear hormone receptor family member nhr-28	*nhr-2*	56	ACAA FFRRSVALNKS YPDFEQLHQE QKKNV LDP MRLANLLVLL PAL QRSVRVIKTEEIDSVW DLNK	7	15
63	WBGene0001398	Uncharacterized protein ZK512.7	*ZK512.7*	64	ILNVDM KYQVNVVRT P VTVYRRILRP AR	6	19
20	WBGene0000185	Putative histone H1.6	*hil-6*	62	LGDQVKKINSN LRALV QTVGTGATGRKPT VK KAATGEKV KIAKPA AKK	11	25
317	WBGene0001206	Uncharacterized protein T26G10.3	*T26G10.3*	68	VAEVILPGR PTTLKADI RRHLNSEKYA NR AEK ELVR	7	39
141	WBGene0000150	14-3-3-like protein 2	*ftt-2*	125	MSDGKEELVNRLAEQ	12	43

					AERYDDMAASMKVTE LGAELSNEERNLLSVA YKNVVGARQQMAKEY REKFLIPKAGAAESKM KGDYYRSQQSYQEAFD IAKDKDSTLIMQLLR		
379	WBGene0000392	Proteasome subunit alpha type-7	*pas-4*	63	NRYDRG STAVGVRGK SIPALQDDRYIANTKQR FT QSPGRVAAEAEEAA SK	9	22
81	WBGene0001600	GYF domain-containing protein C18H9.3	*C18H9.3/C 18H9.2*	56	AS GAFFAGRFNALR SESDEEEEE GWSKAGY FTD ESLRVQRLLDDHN REIEERKNEIIAKE AAD RLEEA TER RLEA ESR VEQ EKSGGLWGASKT VKE FVRQVLDGNILGF R	13	10
324	WBGene00010767	Protein MEF2BNB homolog	*K11B4.2*	58	EIESRSRIISERLQEHTV RSLPGLVNRNCMFYK	8	21
251	WBGene00001673	Guanine nucleotide-binding protein alpha-11 subunit	*gpa-11*	53	MSAADMARKILLLGGP ECGKMVDVGGQRSEQ RKDDGTPTKVFDSCMD VIFK	8	14
262	WBGene0000798	Uncharacterized protein C36E8.1	*C36E8.1*	63	TETLVKRA LLKCVKNL ILMQ KYIPASISKCE QN EEMSKSPRDMLETIRCF SAITRFMTPL MR	12	9
162	WBGene0000848	Serpentine receptor class beta-17	*srb-17*	63	QKINMVKGRIFLPGER QRNSKVQSL ITLR	11	7
380	WBGene0000175	Glutathione S-transferase P 10	*gst-10*	58	GFGEYIRGMLLGQLPC LKMKYVRTIPEALVKS FWE R	5	17
129	WBGene0001384	Protein CLASP-3	*cls-3*	57	TSRMQPKDTSP TRRLS	17	5

					E WLRSRSE NNKKMCL RMCTAQAAKMTPHFT KAISTSL PR		
219	WBGene00007186	Uncharacterized protein B0464.6	*B0464.6*	73	MSPVCKLEQSRITMLE TRSPAVYGKTEVKMLR RSNSIDRRAEFMTRWN SGGSEPTDDNDQMTRL VRQSPNLFR	12	11
208	WBGene0000295	Pre-mRNA-splicing factor CWC22 homolog	*let-858*	61	SKETRSSARS PSRTRT GGAYIPPAKNLVQIVR SKGLLCRLRAILMETE RS ENALDRLDPEFEKL LKM KILADMKMTEED TTSSG RSEE RGGR	12	10
54	WBGene0000997	Uncharacterized oxidoreductase F53C11.3	*F53C11.3*	60	AIATTFAHLGASVAIAA RRCIQQKRAGVENMTK SLASEWAKDSGD AMK SVPVGR	14	17
178	WBGene0000158	Glycine receptor subunit beta-type 4	*ggr-1*	66	K QLADMRAILDFP RD YPQKPPKM KKVAQCV	9	8
100	WBGene0001392	Cytochrome c oxidase assembly factor 7 homolog	*ZK20.4*	78	MHSLFLKRVGNYNR WVDHRLQHNNTKGI RMPDMHLKFYTKR	11	26
237	WBGene0000870	Putative glucosylceramidase 3	*gba-3*	74	SGKISEEAM QEQAER YGMY MLAGKAERYM RTNAAGEA K AELGS L SRSLQ FAIRYADR AKE IMETLR	12	11
289	WBGene0000167	Guanine nucleotide-binding protein alpha-13 subunit	*gpa-13*	61	LQVPEIRSITPASQKSIK ALCRINMVDYKP SER LS VIPLSKVYRCIAIDT Q MMAELLSTVF K	9	17
338	WBGene0000295	Pre-mRNA-splicing factor CWC22 homolog	*let-858*	67	D DGDDQPGTPNSYRSS SARS PSREPLDILRTRT	13	11

					GGAYIPPAKNLV QIVR SKGLLCRS ENALDRLD PEFELLKM KILADMK MTEEDTTSSG RDQEDH REDR		
250	WBGene0001936	Cyclin-dependent kinase 2	*cdk-2*	65	CALKMIRIADFG LARN FSFPSRIFE IVGTPNIKL EETSCLTGNGLDVLRL T AKGALSHR	9	16
265	WBGene0001053	Transthyretin-like protein 2	*ttr-2*	55	LMESDNSFGPGFLD SD DKMASGKA DSHGEFN LSG STKEITGIEP YLVV FHDCKYP GETR	4	39
233	WBGene0000814	Protein FAM50 homolog	*C47E8.4*	64	VGITNKFMANYETVEE VVKSKDSQEAREEHVA KRVG MDPTVDTSFLPD KTG PLFVFDSASD VRI RSWYEK	9	21
372	WBGene0001932	Adenosylhomocysteinase	*ahcy-1*	76	EIILAENEM PGLMAMR Y GPSQPLKGARYPQYL AGIRSK FDNLYGIRANI IVTTTGC KDIVTGKLS DEQAS YLGVPVAGPY KPDHYRY	12	19
60	WBGene0001986	Nuclear RNAi defective-3 protein	*nrde-3*	73	VMGE MGSKPGSTAKT NVWGTAKSGFVKTTKI VDRGHNGGRERYDDR R DGEIDYDERTVSHYQ RLTSI TEYYLQRYNY R QMDAFRFQTNGKFVM PARRPDMHDIL KYFEE KTQFIG FEMSHTGART RLTNLQEKKHGY NPK	16	13
295	WBGene0001340	5'-tyrosyl-DNA	*Y63D3A.4*	88	LKNSSMSDEQKSLLTR	12	17

		phosphodiesterase			MK AVAHIVKMFDVEK DAW EAAGSDNKTKQG FHGAKMR FDRFTLEGR		
432	WBGene0000918	Nucleosome-remodeling factor subunit NURF301	*nurf-1*	60	VCGKSSGRNEMSESFL TRFAQID NFDWVVAKN RKTIFM PQWWNGLGQ TRCYSPMCRNGYLVSA KAKEDEERRHNVILMK IPN ANGGAPRQQD HQ VMRVIKQV LDVMFSQ VCRQKNDSDKFRMNQ RMENEA EQQCGLLTP W R FDA SKLYIQCELC ARVSVQ LTRGNAVFK CA ETMQEVFDK	20	8
273	WBGene0000372	Nuclear hormone receptor family member	*nhr-130*	80	FQF DRDLISATEKRT FGMSRDQPAGS HDVQ LVTKL ALTATSRQN V CEQKTIELDYSWFTKY PK	10	14
571	WBGene0000494	Polycomb protein sop-2	*sop-2*	87	LEGKDPRLQ YYLNR GGRPGYR GAFRGAAR IPPQPLRRTSDYTRTEK YQADL RK	11	9
600	WBGene00002199	Receptor-like tyrosine-protein kinase kin-15	*kin-15*	85	MCLKMRYERIKLEISE DKMIEDETKNGYLLPK IADFGL ARMYDR MKT LKSEAERLEDWIR	14	12
276	WBGene0001405	Lipoamide acyltransferase component of branched-chain alpha-keto acid dehydrogenase	*ZK669.4*	82	MMAARLLGTS SRHL HTSKEWYVKAAVTISC RY DGIVKKVLATPAVR S YEPLKTMTEALKAEL KEFAKNCEQ R	16	16
282	WBGene0000798	Uncharacterized protein	*C36E8.1*	76	RSTANAPKTETLVKRA	13	10

		C36E8.1			LLKCVKNLILMQ KYIP ASISKWLRNFKVLPKE KDMLETIRCFSAITRFM TPL MR		
77	WBGene0002148	Fatty acid-binding protein homolog 9	*lbp-9*	66	MLSAFFKNMPIQT DLV GKYIEN GKCTRVYEK	7	20
299	WBGene0000643	Uncharacterized protein tag-51	*tag-51*	96	WMEAFETKIVEKYARV AAIAASRFANEVVQKN FRYSAKIFQFWK FGSV KET MQKSLKAWSLFE KFDWW PKYQRLRMSN MATKTR	13	16
465	WBGene0001076	Protein MEF2BNB homolog	*K11B4.2*	66	EIESRSRI ISER LQEHTVRS LPGLVNR NCMFYK	10	21
124	WBGene0000692	Vitellogenin-3	*vit-3*	55	ELSDELKDMESDKSET RSKQPLSIR TEAVDAL RLLKVLLPVYKQYFHK VEMD ARRNSEELLVKEE EIV VKVDEKKNK WNR EKTSNEKIRHSKNAR	21	6
498	WBGene0001575	Uncharacterized protein C14B9.3	*C14B9.3*	75	KDENSIRSTEDNRRSD YIPDE RVFNALTIRFSSL H KWDTV SMIMK	10	12
74	WBGene0000133	3'-5' exonuclease eri-1	*eri-1*	81	TAVEVESAETRTDESEK QMDTMTAEQLEMKIIS EF R LYTWMR FNLG QK SG ENMLERLDLSFVGN KDFQLWMRRN DEAAF QEKMPPVSSTLHTEVD LDA VWER	16	19
328	WBGene0001856	cAMP-dependent protein	*F47F2.1*	67	VILSHHKRS PPLLMRLF	12	12

		kinase, catalytic subunit			RR VLLQLKMYASEKF PRSFNL AAKDRDLFAE W		
413	WBGene0000022	ATP synthase subunit beta, mitochondrial	*atp-2*	80	GQPVADTGDP IKIPVG PETLGRIMNVIGEP IDE RGPIASKEGNDLYHEM IEGGVIDLKGKIPSAVG Y QPTLATDMGSMQER GIAELAIYPAVDPL DST SRIMDPN VVGQNHYD IA RGVQK	15	25
139	WBGene0001127	Probable thymidylate kinase	*R53.2*	63	RGLL IVFEGLDRLVE SINKK EIDMDEHALH L LFSADR	7	16
270	WBGene0000798	Uncharacterized protein C36E8.1	*C36E8.1*	78	A LLKCVK VMPHFTRP SVTVA GYMRTK LNDK WLRITGDKVLPKEKAK YVKCFSAITRSEKSAEA VSSSEG LDFLDEDDAM MMGGSSGYR	9	13
122	WBGene0000692	V-type proton ATPase subunit C	*vha-11*	72	GA NDAWDKYLIPDLK FQWE GAKNALASMDR DLAD LVKKVIDEFK NT ARENKQYAP LIR	9	15
364	WBGene0000369	Nuclear hormone receptor family member nhr-100	*nhr-100*	67	HYGAMS CNGCKGFFR EIIAHMLYEEDRVLNW EEPYNKFNW CRTFTLT IDW FETLPEYRFSLMPV GWLWYAYKITESFMA DVVNSMKELEMD ETE MVLLKALCEYIRMK	13	24
125	WBGene0000914	Nuclear cap-binding protein subunit 2	*ncbp-2*	67	EISAYR YQGTVRDQE TALR A GDVRRVIMGL DRQYGRGK	11	24

137	WBGene0000472	Protein sax-3	*sax-3*	70	VNSGKNGKLAMLRAAL KV TTKM DMAIAEK TI NSTAVRIRMLNLTTLR NITTNERVAA RSNGGV GVSHG TSEVIMNQDT LEKSDSPPHT DVSYVQ LHSSDGTGSSKERRTPP NKFRS IPR	16	9
575	WBGene0001631	Protein EFR3 homolog	*C32D5.3*	78	NGLCCCTPC KPRYRLV DS IYPRAVTDGL LYSN MQKAVIEPV LKKHI GE ITYMLLRLFVPFN FNM NGRKES PSGYKNTIFSI VNP PKAYANATFD PIE EGRKNGEMTR	15	13
48	WBGene0000442	60S ribosomal protein L13a	*rpl-16*	74	L ASIVAKYMSFLR CNI PARGNEALKSLHAPSAS RFR KFCVVGRNAAPKI QYQK	9	27
308	WBGene0000186	Structural maintenance of chromosomes protein 1	*him-1*	55	LKVEMTKEEINAQKKL QKQQNM LTLKKM LA ESKEHQERVKETEEET EIKKPAGVKSNEVAD R IFADFCTRVEVEKEKKI AQVAMKLE SLLTKKD VDDDDGVRNVSKM NA PNLK	20	9
281	WBGene0000675	Paramyosin	*unc-15*	82	MELQELLIVSLESKS PS QAAFGAPF GSMSVAD LGSLTRLEDAEGTTDSQ IESNRKRLEDAERERSQ LQSQLHQVQ LELDSVR ISQLEKQVGELKVRIEM ERRKQ SEQLK IDALRK LE TRIRET QNALRHRS	15	15

					SVVTGK		
86	WBGene0001006	Dehydrogenase/reductase SDR family member 4	*dhrs-4*	45	PSNCRRGIGLAIAERLV D FTLQKTTLVG LTRAL AMGLA K	7	1
115	WBGene00020462	Probable protein ariadne-2	*T12E12.1*	80	FARILLQANHWDVDKC HIDAK PEPKRSHVES R FLLRDMVN SHPHLK RVTCMQCHTSFCVKC GADYHAPTSCETIKQW MTKMEEELR	11	17
218	WBGene00000452	Homeobox protein ceh-31	*ceh-31*	59	TIFTDKMELAHRT WYQ NRNASVSKKESDEDE	5	11
517	WBGene00000292	F-actin-capping protein subunit alpha	*cap-1*	91	MSEISDAEKNFCNGRW RSE WNVPVGDGKS GS QEMKRQL PVTRAKMD WN K	8	17
76	WBGene00006364	Liprin-alpha	*syd-2*	48	ERDMMKAE RNNTRAR ATYAAEA EEAMASNA I NGSISSESAN RVHSLQ ERIY DQAERQQLYN A VAE QLERYDI YGNPQF VDDRMVRMLEHLSK	14	8
194	WBGene00011145	Uncharacterized calcium-binding protein R08D7.5	*R08D7.5*	65	CTEQKPNLL KLSQLKD MDVDEL WNALQSKDD E GDSTEDKVTT EMRE LG ETLADADFDE MIR	9	41
506	WBGene00015161	Putative acid phosphatase 5	*pho-5*	71	RYGSWLGEKSSDYNRT LMSA QANMAGLFPP K YPIAGGLMW QPIPVHT ISK PTDKELYEEASCPT AEIEMN AQWKDNLFC EKVF HKNETDIDRKYF PEDWE AECGLK	9	26
287	WBGene00016321	Tubulin polymerization-	*tppp-1*	65	WDA FTKDAGVLDNKA	7	33

		promoting protein homolog			ITGTMTGIA FSKVTGP KSGRADTTE NTGYVG YKN KDSYDKTHGK		
462	WBGene00006537	Tubulin beta-2 chain	*tbb-2*	83	IDVYNEANNGKYVPRA VLVDLEP GTMDSVRFP QLNADLRKLAVNMVP F PRYL TVAAMFRTAV CDIPPRG LKISEQFTAM FR	18	18
103	WBGene00018849	FACT complex subunit spt-16	*spt-16*	63	DLFFQRVPTVSTLLRD KLIDHIKVQQA LAKLS DVYKLA ETLNKKSNV SYKFPQ DADVQKLNP KLKRPHSS KSGPSHKR	13	7
341	WBGene00000066	Actin-4	*act-4*	80	CDDEVAALV VDNGSG MCKA GFAGDDAPRA V FPSIVGRPR HQGVMVG MGQKDSYVGDEAQ SK EHPVLLTEAP LNPK S YELPDGQVITVGNER	10	25
