# Supplementary material for: Global Proteomics Revealed Klebsiella pneumoniae Induced Autophagy and Oxidative Stress in Caenorhabditis elegans by Inhibiting PI3K/AKT/mTOR Pathway during Infection
Source: Front Cell Infect Microbiol. 2017 Sep 6;7:393. doi: 10.3389/fcimb.2017.00393 (PMC5592217; doi:10.3389/fcimb.2017.00393)
Supplement: Supplementary Table 3 — List of proteins identified in nematodes exposed to K. pneumoniae for 36 h by MALDI-TOF/TOF analysis. [file Table3.PDF]

Spot No	Wormbase ID	Protein	Gene	Mascot score	Peptides identified	No of peptides matched	% of sequence coverage
643	WBGene00002199	Receptor-like tyrosine-protein kinase kin-15	*kin-15*	38	MCLKMRYERSNPEVRL EISEDKMIEDETKNGYLL PKNTIRMYDR MKLEDWI R	14	10
366	WBGene00016677	Uncharacterized protein C45G9.6	*C45G9.6*	26	NAPEPGKINSGCERISME LKIT KVVVKEKV IPKM GESCSGRYDMFKKNFIL R TL	13	9
273	WBGene0001384	Protein CLASP-3	*clasp-3*	26	TSRMQPKSLVIHGEKDT SPT RRIRSPLKLSEWLRS RSE NNKKMCLRMCTAQ AAKMTPHFTKAISTSL P R	14	6
220	WBGene00009885	Molybdenum cofactor biosynthesis protein 1	*F49E2.1*	25	SSTE EIVKQLTVPL RML SNSEVLRGYYPKVKLSD SPN DTTKITADGNL KH FFISQIRQVDVSQKD TST RTAVAR	10	12
549	WBGene00003766	Neuropeptide-like protein 28	*nlp-28*	25	GMYGGYGRGMYGGYG RGMYGGWGK	8	36
621	WBGene00012276.	Histone H3.3	*his-74*	28	KALATKAAR SAIVTGSV KSTELLLRKLPFQR RVTI MPK	10	27
58	WBGene00000917	Dauer abnormal formation protein 25	*daf-25*	28	TTTEEAPKTKEQAAQRC DVNDKLLMDSGARILY VIDRALDVMLRKVCSFL K	13	13
254	WBGene00008920	Probable elongation factor 1-gamma	*eef-1G*	80	TGKLYGNKTVTL AGDA APADKTVEQYK K SIVN VTRTVVNQPAVKEEKSK	9	14

					LDAKSD ATK		
344	WBGene00021486	Fatty acid-binding protein homolog 9	*lbp-9*	32	MLSAFFKNMPIQTDLVG KDSRIERYIEN GK	7	19
381	WBGene0000670	Probable ubiquitin-conjugating enzyme E2 7	*ubc-7*	47	EQSSLLLKK QLADMRR AILDFPRDYP QKPPKMK KVAQCVR	10	24
573	WBGene00007403	SET domain-containing protein 3	*set-3*	23	VEVPSFRVTVKWDK IM AERSAKVLEKPKKFVEN AKARMLWK	11	8
322	WBGene00013986	Uncharacterized protein ZK512.7	*ZK512.7*	27	ILNVDM KYQVNVVRTP VTVYRRILRP AR	5	19
568	WBGene00004702	Probable splicing factor, arginine/serine-rich 5	*rsp-5*	30	IPYNARERDVERFLK IN N ISMKLVVEMARSRTPP RASRSRSPLPAKDRSPSP GSPKD	15	27
92	WBGene00019800	Single-stranded DNA-binding protein,	*mtts-1*	20	SLSTISKMAAEQPSKHA VSVFGK QAEILSKENPD QH	8	21
60	WBGene00006920	V-type proton ATPase subunit C	*vha-11*	32	GA NDAWDKYLIPDLKN ALASMDREWE QKKVIDEFK NTARENKD KL MAEKQRQYAP LIR	13	15
415	WBGene00004726	Spindle assembly abnormal protein 4	*sas-4*	31	NAKPDEDTRSVASIRF N LNENKILTAKEKVERDL TKSDMEE LEAQIRDLQS QLSDQKENELR KTISDL R	11	6
656	WBGene00001586	Glycine receptor subunit beta-type 4	*ggr-1*	28	MHSLFLKRVGNYNRRL QHNNTKGI RMPDMHLK FYTKR	11	8
442	WBGene00001608	Uncharacterized protein R07B1.8	*R07B1.8*	22	ERFLRMLKHGRMLDMLK CGEQNKLAKNYKKA LED EK	10	10

400	WBGene00003526	Zinc metalloproteinase nas-7	*nas-7*	30	EELFGKN GVSRAAKGA LDQFGKNGF PTMLPKV KSATIGNARSVTA PFAR	8	12
363	WBGene00011499	Uncharacterized protein T05G5.4	*T05G5.4*	40	VSILMD KADLYEKNQS RKDFEQYLNFR	5	18
69	WBGene00044763	Uncharacterized protein ZK688.12	*ZK688.12*	21	KGDVESTKNRYTYKNK LSNEKMGIRNQNMGEK K	7	19
265	WBGene00004915	Protein CLASP-3	*cls-3*	23	TISKNNKQIKPKAGK TD GEEKRILGLV LVRDDSVR	17	7
269	WBGene00006920	V-type proton ATPase subunit C	*vha-11*	31	YMSFLRAYEGVPAKSLH APSASRFRIA QYQK	12	15
46	WBGene00002583	Target of rapamycin homolog	*let-363*	20	SRITE NRYANYLLKLAA SHLSRYIFNAVRKLVLE RMIMMRLEQHSAKMIA QLAKQSPKREAA LRVM ESIQK YG VEEKEMSVL KKGRWYEKKQLDHKQL YQD DHKNAGTVLEKFP ERVPFRLVEGMKK	22	4
466	WBGene00008474	Nuclear hormone receptor family member	*nhr-174*	32	YLCEKFDYCVKNLVRQ RKSSMSPNKE AEKDVS KILKLAAEICK FINQVK	13	15
508	WBGene00002087	Probable insulin-like peptide beta-type 1	*ins-4*	22	QPSMDTSKADRILRRVP AGEVR	4	27
311	WBGene00016677	Uncharacterized protein C45G9.6	*C45G9.6*	29	NAPEPGKISMELKMSDIP SEHVIAGQHDGEAVRIT KVVVKEEKEKV IPKMG ESCSGRNVTVCRYDMF KK	10	12
59	WBGene00018849	FACT complex subunit spt-16	*spt-16*	26	VPTVSTLLRDKLIDHIK VQQA LAKLSDVYKLA	16	8

					ETLNKCDEVVKLKSNVI<br>K EEQENRMLGRGQRFP<br>Q DADVQKLNPKLK		
184	WBGene00006926	Vitellogenin-2	*vit-2*	57	RAVVNMISFNPIAPRNEI<br>EKFPLDKKMHLIKLVRIF<br>RQAAW LAAGSVVREAI<br>DALRLLKDTMPRMLAL<br>WRVAIVCSKVLS FTRIQ<br>AVAGMKNVQVPRVYLP<br>SIAQRFTCLQRHNRDAS<br>RLLKVPTAC VAY	21	8
74	WBGene00000452	Homeobox protein ceh-31	*ceh-31*	70	SMLDLRTIFTDKYLSVQD<br>RMELAHRNASVSKKESD<br>EDE	8	14
476	WBGene00021629	CTD small phosphatase-like protein 3	*scpl-1*	33	MSNVTPKLRPHLRKVY<br>ANKNHIRHRDLTILGRN<br>DTELL KYRLR	7	12
505	WBGene00001857	Putative histone H1.6	*hil-6*	24	LGDQVKALVQTVGTGA<br>TGRAATGEKVKIAKPAA<br>KKAAAK PAKKTAALK	10	25
382	WBGene00000292.	F-actin-capping protein subunit alpha	*cap-1*	21	SEISDAEKSFKYDHVRN<br>FCNGRRQLPVTRAKMD<br>WN K	7	12
567	WBGene00001414	Sperm vesicle fusion protein fer-1	*unc-22*	32	VKSASTP VAK AGSP STRK<br>SKSPAPQA KK TDSPPK<br>TEFKMEK S SIDMRRESVQ<br>EILEK QSL FPGKK DTEVIEK<br>NPQLADTG K LKVG ETIK GK<br>HIMK FTITL K GKYIEV GK<br>VNGLRNK AHIDGLK<br>KGQTYQFR ADLE WK LNN<br>EDGK VKALN K TMVAKPR<br>DMF VAQR IVAT NESGK		4

					ESVVLK GDTGVYK SDS GPVKIK K TYEFR DNRY R EPEF TVDKLR VVAINA AGK WFKGC K GETIELK AYPQGEA ETVRK DDRELKQSVK YMK FEPLEPMK VSSF RPR GSEKIEK Y AAALLLV		
281	WBGene00022739.	HEAT repeat containing protein 1	*toe-1*	24	HL TVEKR IGVA GLEQMK SPMKSFR ATEW LNGEK Q IDGGSVK DCLRLIVAAA KHTPNSVVK LIELAR LGGDKPA K TAQT LPR NA LPLGK MCQNSAYTL QQSL GGNK T GIHHRLSLIR R	18	7
486	WBGene00000452	Homeobox protein ceh-31	ceh-31	26	MNNKGR RD MTPTR SVEGEALKQ MMRHK STRH VK TTEYVR R QFQKVR FNANYK NVGLNVR	9	12
185	WBGene00014243	DDB1and CUL4associated factor	*dcaf-1*	30	EIYPLR SLE EQKK NLDDSNSK YLTSLLVHR K G GIPALLK SSSA SKR T LESEIGGISA R LMISPA R FRN WK LGEALQR IFSSFR DLRMGIR GAEAERR	19	5
274	WBGene00000937.	Lariat debranching enzyme	*dbr-1*	22	K H H P D R G L L E M D R N K G V D N D E V I K L Y L I R K D A A E Q E K	10	9
