# Supplementary material for: Global Proteomics Revealed Klebsiella pneumoniae Induced Autophagy and Oxidative Stress in Caenorhabditis elegans by Inhibiting PI3K/AKT/mTOR Pathway during Infection
Source: Front Cell Infect Microbiol. 2017 Sep 6;7:393. doi: 10.3389/fcimb.2017.00393 (PMC5592217; doi:10.3389/fcimb.2017.00393)
Supplement: Supplementary Table 4 — List of proteins separated by liquid phase IEF and identified by nano LC-MS/MS analysis. [file Table4.PDF]

Control

Accession No	Protein	Gene	Score	Coverage	Unique peptide	MW (kDa)	Calc.PI
Q95ZL1	M03F4.2b	*act-4*	7.86	32.23	2	37.3	5.55
Q18066	Disorganized muscle protein	*dim-1*	4.80	29.69	1	71.8	7.96
Q19790	F25H8.5a	*dur-1*	5.20	18.46	2	79.5	6.20
Q93576	Nucleoside diphosphate kinase	*ndk-1*	47.43	26.14	3	17.1	7.50
Q23621	Glutamate dehydrogenase	*gdh-1*	43.87	33.21	6	58.8	7.31
Q10454	Probable arginine kinase F46H5.3	*F46H5.3*	41.11	33.59	3	44.1	7.25
Q27245	Putative aminopeptidase W07G4.4	*lap-2*	39.20	31.42	5	56.1	7.05
Q9XXK1	ATP synthase subunit alpha, mitochondrial	*H28O16.1*	38.92	26.95	5	57.8	8.94
Q20780	F54D8.3a	*alh-1*	36.57	38.63	7	55.0	7.53
O17214	Probable fumarate hydratase, mitochondrial	*fum-1*	33.74	18.36	3	53.6	7.96
P17329	Glyceraldehyde-3-phosphate dehydrogenase 2	*gpd-2*	33.45	33.72	6	36.4	7.27
Q95ZL1	M03F4.2b	*act-4*	31.57	22.59	3	37.3	5.55

P34559	Probable enoyl-CoA hydratase, mitochondrial	*ech-6*	30.50	26.39	4	31.2	8.38
O17953	Dihydrolipoyl dehydrogenase,	*did-1*	26.81	23.64	3	52.6	7.68
P54216	Fructose-bisphosphate aldolase 1	*aldo-1*	24.86	9.56	2	39.2	6.73
Q10657	Triosephosphate isomerase	*tpi-1*	21.64	22.27	3	26.6	6.70
P34455	Probable aconitate hydratase, mitochondrial	*aco-2*	18.84	13.77	4	84.0	8.03
O17626	C31C9.2	*C31C9.2*	18.76	14.29	3	34.7	6.80
G5ED31	VW06B3R.1a	*ucr2.1*	18.07	7.32	2	42.7	8.07
Q19842	Propionyl-CoA carboxylase alpha chain, mitochondrial	*pcca-1*	17.42	26.10	4	79.7	7.74
Q18599	C44B7.10	*C44B7.10*	17.29	19.11	3	50.7	8.54
P41977	Superoxide dismutase [Mn] 2, mitochondrial	*sod-3*	16.52	19.27	2	24.6	8.38
P36573	32 kDa beta-galactoside-binding lectin	*lec-1*	15.99	39.43	3	31.8	6.60
P31161	Superoxide dismutase [Mn] 1,	*sod-2*	13.36	15.84	1	24.5	7.94
Q20684	F52H3.7a OS=Caenorhabditis elegans GN=lec-2 PE=1 SV=2 -	*lec-2*	12.64	11.41	4	131.9	4.37

	[Q20684_CAEEL]						
Q11176	Actin-interacting protein 1	*unc-78*	12.45	16.04	4	65.3	7.08
H2L0N9	Y37E3.17a	*CELE_Y 37E3.17*	12.38	14.34	1	91.8	7.46
P29691	Elongation factor 2	*eef-2*	12.25	21.71	3	94.7	6.52
P46562	Putative aldehyde dehydrogenase family 7 member A1 homolog	*alh-9*	11.46	18.83	2	57.0	6.70
Q86DB5	C46F11.2b	*gsr-1*	10.75	9.37	2	49.8	6.67
Q17994	Aspartate aminotransferase	*got-2.2*	10.28	27.05	1	45.6	9.16
Q27888	L-lactate dehydrogenase	*ldh-1*	9.49	26.43	2	36.0	6.90
O16264	Phosphatidylethanolamine-binding protein homolog F40A3.3	*F40A3.3*	9.07	33.48	1	24.1	8.73
U4PR97	Y66H1B.2n	*fln-1*	8.60	14.40	3	225.0	6.25
P34385	Probable methylcrotonoyl-CoA carboxylase beta chain, mitochondrial	*F02A9.4*	7.33	18.09	2	66.5	8.31
G5ED44	Delta(9)-fatty-acid desaturase fat-5	*fat-5*	7.32	11.71	1	38.4	8.06

O45148	W02F12.5	*dlst-1*	7.30	20.73	1	49.8	9.09
P34697	Superoxide dismutase [Cu-Zn]	*sod-1*	7.14	16.11	2	18.7	6.64
U4PE16	Catalase	*ctl-3*	6.45	20.49	1	56.9	6.87
O44549	K06A5.6	*acdh-3*	6.23	18.14	2	45.7	7.77
O44893	T20H9.6	*CELE_T20H9.6*	6.22	15.06	1	83.3	6.52
O01685	C32F10.8a OS=Caenorhabditis elegans GN=C32F10.8 PE=1 SV=2 - [O01685_CAEEL]	*C32F10.8*	6.22	14.29	1	55.8	7.40
P02566	Myosin-4	*unc-54*	6.15	15.03	1	224.6	5.74
Q94055	Serine--pyruvate aminotransferase	*CELE_T14D7.1*	6.08	21.48	1	43.9	8.19
G8JY45	Fructose-bisphosphate aldolase	*aldo-2*	6.06	28.96	1	28.0	7.05
Q22288	Transthyretin-like protein 15	*ttr-15*	6.03	22.31	2	14.5	7.74
O45679	Bifunctional L-3-cyanoalanine synthase/cysteine synthase	*cysl-2*	5.32	24.63	2	36.1	7.68
G8JY74	R03G5.1d	*eef-1a*	5.13	12.59	2	47.1	9.00
Q95XJ0	Y69A2AR.18a	*CELE_Y69A2AR.18*	4.70	14.05	1	32.4	9.04

O44156	Proteasome subunit alpha type-1	*pas-6*	4.53	29.23	1	28.3	7.02
O17759	F01G10.1	*tkt-1*	4.40	20.39	1	66.0	6.64
Q9NAK4	Y38E10A.7	*lips-15*	4.39	14.69	1	29.9	6.52
P98080	Cytochrome b-c1 complex subunit 1, mitochondrial	*ucr-1*	4.36	9.98	1	51.7	6.54
P91466	T20D4.9	*CELE_T20D4.9*	4.22	2.93	1	50.8	5.99
O62512	ZK550.3	*CELE_ZK550.3*	4.05	21.76	1	90.3	6.57
Q19328	F10G7.2	*tsn-1*	3.85	13.35	1	100.7	7.75
H9G351	ZC101.2k	*unc-52*	3.72	8.80	1	250.2	5.33
G5ED07	Protein disulfide-isomerase	*pdi-3*	3.38	14.55	1	54.9	6.33
Q21243	K05F1.3	*accdh-8*	3.33	16.75	1	44.5	8.24
Q18943	D1054.10	*CELE_D1054.10*	3.01	38.30	1	20.6	8.84
Q93714	Probable isocitrate dehydrogenase [NAD] subunit alpha, mitochondrial	*idha-1*	2.83	22.91	1	38.4	7.39
Q09657	ZK1320.9	*CELE_Z*	2.73	12.71	1	51.3	8.62

		K1320.9					
O45815	T25C8.2	*act-5*	2.60	6.13	1	41.8	5.68
O17744	E03H4.4	*CELE_E03H4.4*	2.58	8.45	1	93.7	6.65
P13508	Protein glp-1	*glp-1*	2.53	9.50	1	144.0	5.72
C7IVQ8	F19B10.4	*CELE_F19B10.4*	2.50	14.88	1	39.2	5.10
A0PCJ1	T21D12.14	*CELE_T21D12.14*	2.49	26.36	1	12.5	5.64
Q10663	Bifunctional glyoxylate cycle protein	*id-1*	2.48	14.88	1	108.6	6.89
Q9BKU4	Mitochondrial prohibitin complex protein 1	*phb-1*	2.48	22.91	1	30.0	7.56
Q9XVH2	W02B8.4	*mltn-4*	2.47	16.37	1	82.9	9.60
O44548	K06A5.4	*knl-2*	2.41	15.96	1	101.1	9.22
H2KY92	F42G9.6a	*dagl-2*	2.38	9.89	1	73.7	6.29
P52713	Probable methylmalonate-semialdehyde dehydrogenase [acylating], mitochondrial	*alh-8*	2.34	23.14	1	56.4	7.65

G5EFP3	F21G4.2	*mrp-4*	2.33	5.72	1	177.5	7.06
G4SL51	M01E11.4c	*pqn-52*	2.29	8.82	1	62.6	6.04
G5ED06	C14B4.2	*C14B4.2*	2.28	10.19	1	196.3	6.37
Q93572	60S acidic ribosomal protein P0	*rpa-0*	2.25	30.13	1	33.8	6.71
Q23511	ZK524.3a	*lars-2*	2.25	26.31	1	98.2	8.79
Q9U208	Y57A10A.15	*polg-1*	2.23	6.72	1	121.8	6.30
G5EFP6	Y119D3B.3	*srz-9*	2.21	16.73	1	32.2	9.20
P11141	Heat shock 70 kDa protein F, mitochondrial	*hsp-6*	2.20	11.11	1	70.8	6.20
G3MU38	C52E4.2c	*mif-2*	2.18	24.11	1	15.2	9.76
O01600	T10E9.4	*CELE_T10E9.4*	2.16	18.64	1	71.1	7.94
G5EF35	F59A1.13	*CELE_F59A1.13*	2.14	4.86	1	54.1	7.40
O16228	Glutathione-independent glyoxalase DJR-1.2	*djr-1.2*	2.09	21.51	1	19.5	6.55
Q19749	Dihydrolipoyllysine-residue acetyltransferase component of pyruvate dehydrogenase complex, mitochondrial	*F23B12.5*	2.06	11.64	1	53.4	8.25

***K. pneumoniae* infected**

Accession No	Protein	Gene	Score	Coverage	Unique peptide	MW (kDa)	Calc.PI
P02566	Myosin-4	unc-54	56.57	22.57	10	224.6	5.74
Q27527	Enolase	enol-1	29.36	42.86	7	46.6	5.86
P02567	Myosin-1	myo-1	16.13	18.42	3	223.2	6.07
P46562	Putative aldehyde dehydrogenase family 7 member A1	alh-9	12.53	17.89	2	57.0	6.70
Q6A8K1	M03F4.2c	act-4	11.11	30.94	5	40.4	5.83
P12844	Myosin-3	myo-3	9.33	10.82	1	225.4	5.66
H2L0I2	ZC8.4a	lfi-1	7.16	13.81	2	273.7	6.15
O44400	Protein F37C4.5	F37C4.5	7.16	15.65	2	61.4	5.68
P34697	Superoxide dismutase [Cu-Zn]	sod-1	6.83	16.67	2	18.7	6.64
P12845	Myosin-2	myo-2	6.60	14.84	1	222.9	6.34
P98080	Cytochrome b-c1 complex subunit 1,	ucr-1	6.50	20.81	3	51.7	6.54
O16228	Glutathione-independent glyoxalase DJR-1.2	djr-1.2	6.07	39.78	1	19.5	6.55

P50140	Chaperonin homolog Hsp-60,	hsp-60	6.04	17.43	1	60.1	5.40
Q19626	Probable V-type proton ATPase subunit B	vha-12	5.96	8.96	1	54.7	5.48
Q94272	K10C2.4	fah-1	5.84	16.75	1	46.0	6.13
Q9TYL2	Y25C1A.13	CELE_Y25C1A.13	5.83	17.51	1	33.0	7.31
Q19420	Inositol monophosphatase ttx-7	ttx-7	5.65	18.60	1	31.0	5.82
Q19133	F07A11.5	CELE_F07A11.5	5.47	8.92	1	33.0	5.99
P54216	Fructose-bisphosphate aldolase 1	aldo-1	4.79	8.20	1	39.2	6.73
Q9XXK1	ATP synthase subunit alpha,	H28O16.1	4.63	21.56	2	57.8	8.94
O76840	Papilin	mig-6	4.61	5.81	1	237.4	5.06
Q23621	Glutamate dehydrogenase	gdh-1	4.58	11.75	1	58.8	7.31
Q10657	Triosephosphate isomerase	tpi-1	4.46	17.81	2	26.6	6.70
O17953	Dihydrolipoyl dehydrogenase,	dld-1	4.35	9.49	2	52.6	7.68
P29691	Elongation factor 2	eef-2	4.34	17.14	1	94.7	6.52
Q93244	Cysteine synthase 1	cysl-1	4.31	20.82	1	35.9	5.96

Q21443	Lamin-1	lmn-1	4.23	17.14	1	64.0	5.59
P27604	Adenosylhomocysteinase	ahcy-1	4.05	23.57	1	47.5	6.25
Q06561	Basement membrane proteoglycan	unc-52	3.93	5.07	1	368.8	4.89
A0A061ACH9	alpha-1,2-Mannosidase	C47E12.3	3.65	7.23	1	56.1	5.17
P34455	Probable aconitate hydratase,	aco-2	3.25	9.52	1	84.0	8.03
Q86GU3	C32F10.8b	C32F10.8	3.06	14.57	1	38.7	7.71
P34559	Probable enoyl-CoA hydratase,	CELE_Y24D9A.8	2.43	13.19	1	31.2	8.38
Q966C7	Transaldolase	CELE_Y24D9A.8	2.33	29.47	1	35.3	6.44
O17626	C31C9.2	C31C9.2	2.32	16.46	1	34.7	6.80
Q27488	Proteasome subunit alpha type-2	pas-2	2.31	7.79	1	25.3	6.05
O01812	Fatty acid-binding protein homolog 6	lbp-6	2.28	9.63	1	15.6	7.40
Q2HQL4	Glutamine synthetase	gln-3	2.26	12.99	1	43.2	6.46
Q10121	RutC family protein	C23G10.	2.24	43.27	1	18.0	5.60

		2					
P92005	M04G12.2	cpz-2	2.22	10.49	1	53.1	5.03
P18948	Vitellogenin-6	vit-6	2.20	10.12	1	193.2	7.23
Q19749	Dihydrolipoyllysine-residue acetyltransferase component of pyruvate dehydrogenase complex, SV=1 - [ODP2_CAEEL]	F23B12.5	2.17	6.31	1	53.4	8.25
O16284	Cysteine synthase	cysl-4	2.13	15.73	1	36.3	8.85
Q21742	R05F9.6	CELE_R05F9.6	2.12	5.81	1	61.7	6.49
Q18817	C54C6.2	ben-1	2.10	15.32	1	49.4	4.94
G5EDD1	VW06B3R.1b	ucr2.1	2.10	8.73	1	44.4	8.76
Q7Z1Q3	Y69F12A.2a	alh-12	2.08	4.61	1	53.2	5.44
Q20829	Alpha-mannosidase	aman-1	2.08	5.34	1	109.3	6.43
Q18066	Disorganized muscle protein 1	dim-1	2.07	11.56	1	71.8	7.96
Q09567	F48E8.3	CELE_F48E8.3	2.04	15.62	1	51.7	5.83
V6CLN5	ZC123.3a	zfh-2	2.03	9.47	1	180.5	6.34
Q2EEM8	JC8.14	ttr-45	2.02	27.86	1	15.5	7.14

H9G341	H12D21.7c	mpst-3	2.00	15.87	1	34.5	7.43
P36573	32 kDa beta-galactoside-binding lectin	lec-1	1.99	17.56	1	31.8	6.60
O45402	F23B2.3	delm-1	1.99	11.66	1	70.6	6.55
V6CJB8	F54E2.3h	ketn-1	1.98	5.91	1	549.9	5.71
O44893	T20H9.6 OS=Caenorhabditis elegans	CELE_T 20H9.6	9.18	11.67	9	83.3	6.52
P29691	Elongation factor 2	eef-2	4.90	13.85	10	94.7	6.52
Q19404	Yes-associated protein homolog 1	yap-1	4.58	5.66	4	50.7	7.01
G5ED44	Delta(9)-fatty-acid desaturase fat-5	fat-5	4.21	9.31	3	38.4	8.06
G5ED44	Delta(9)-fatty-acid desaturase fat-5	fat-5	4.21	9.31	3	38.4	8.06
Q19790	F25H8.5a	dur-1	3.32	15.90	9	79.5	6.20
O76840	Papilin	mig-6	3.30	5.35	10	237.4	5.06
Q23621	Glutamate dehydrogenase	gdh-1	3.23	22.57	10	58.8	7.31
Q9GYT7	F35F10.10	CELE_F 35F10.10	2.81	17.75	10	79.3	7.24
Q95ZL1	M03F4.2b	act-4	2.55	19.88	5	37.3	5.55

Q8IA49	Malate dehydrogenase	mdh-1	2.46	10.62	2	29.1	9.09
P17329	Glyceraldehyde-3-phosphate dehydrogenase 2	gpd-2	2.41	8.21	2	36.4	7.27
O17953	Dihydrolipoyl dehydrogenase, mitochondrial	dld-1	2.34	10.91	6	52.6	7.68
